# *In Silico*, Molecular Docking and *In Vitro* Antimicrobial Activity of the Major Rapeseed Seed Storage Proteins

**DOI:** 10.3389/fphar.2020.01340

**Published:** 2020-09-08

**Authors:** Mahmudur Rahman, Jessica J. Browne, Jacoba Van Crugten, Md. Fahim Hasan, Lei Liu, Bronwyn J. Barkla

**Affiliations:** ^1^ Southern Cross Plant Science, Southern Cross University, Lismore, NSW, Australia; ^2^ School of Health and Human Sciences, Southern Cross University, Bilinga, QLD, Australia; ^3^ Pharmacy Discipline, Khulna University, Khulna, Bangladesh

**Keywords:** rapeseed, napin, cruciferin, plant antimicrobial peptide, *in silico* molecular docking, seed storage protein, 2S albumin, 12S globulin

## Abstract

**Background:**

In addition to their use as an edible oil and condiment crop, mustard and rapeseed (*Brassica napus* L., *B. juncea* (L.) Czern., *B. nigra* (L.) W.D.J.Koch, *B. rapa* L. and *Sinapis alba* L.) have been commonly used in traditional medicine for relieving pain, coughs and treating infections. The seeds contain high amounts of oil, while the remaining by-product meal after oil extraction, about 40% of seed dry weight, has a low value despite its high protein-content (~85%). The seed storage proteins (SSP) 2S albumin-type napin and 12S globulin-type cruciferin are the two predominant proteins in the seeds and show potential for value adding to the waste stream; however, information on their biological activities is scarce. In this study, purified napin and cruciferin were tested using *in silico*, molecular docking, and *in vitro* approaches for their bioactivity as antimicrobial peptides.

**Materials and Methods:**

The 3D-structure of 2S albumin and 12S globulin storage proteins from *B. napus* were investigated to predict antimicrobial activity employing an antimicrobial peptide database survey. To gain deeper insights into the potential antimicrobial activity of these SSP, *in silico* molecular docking was performed. The purified *B. napus* cruciferin and napin were then tested against both Gram-positive and Gram-negative bacteria for *in vitro* antimicrobial activity by disc diffusion and microdilution antimicrobial susceptibility testing.

**Results:**

*In silico* analysis demonstrated both SSP share similar 3D-structure with other well studied antimicrobial proteins. Molecular docking revealed that the proteins exhibited high binding energy to bacterial enzymes. Cruciferin and napin proteins appeared as a double triplet and a single doublet, respectively, following SDS-PAGE. SDS-PAGE and Western blotting also confirmed the purity of the protein samples used for assessment of antimicrobial activity. Antimicrobial susceptibility testing provided strong evidence for antimicrobial activity for the purified napin protein; however, cruciferin showed no antimicrobial activity, even at the highest dose applied.

**Discussion:**

*In silico* and molecular docking results presented evidence for the potential antimicrobial activity of rapeseed cruciferin and napin SSP. However, only the *in vitro* antimicrobial activity of napin was confirmed. These findings warrant further investigation of this SSP protein as a potential new agent against infectious disease.

## Introduction

Mustard and rapeseed (*Brassica napus* L., *B. juncea* (L.) Czern., *B. nigra* (L.) W.D.J.Koch, *B. rapa* L. and *Sinapis alba* L.), and the low glucosinolate, low erucic acid variety, Canola are the second most abundant oilseed crops in the world (Rahman et al., 2018b). Following extraction of the oil, the residual seed meal or oil free press cake, which is high in protein, has potential to be developed into a low cost by-product (Joehnke et al., 2018). The major proteins within the mature harvested *Brassica* seeds are the 12S globulin-type cruciferins, 2S albumin-type napins, and the oil-body proteins, oleosins (Ramlan et al., 2002; Von Der Haar et al., 2014).

Cruciferins, also classified as 11S globulins based on their sedimentation coefficient, are salt soluble neutral glycoproteins (Ren and Bewley, 1999) with molecular weights ranging from 20 to 40 kDa (Von Der Haar et al., 2014), and an isoelectric point (pI) of 7.2 (Stone et al., 2014). They comprise up to 50–70% of the total seed protein (Ramlan et al., 2002; Ebrahimi et al., 2009; Kasprzak et al., 2016). These comparatively larger SSP proteins are composed of two polypeptide chains; the α-chain of 30 kDa and a β-chain of 20 kDa, held together by a disulphide bond (Wu and Muir, 2008; Stone et al., 2014).

The other abundant SSPs are the napins; these are water soluble low-molecular weight basic proteins classified as 2S or 1.7S proteins (Ren and Bewley, 1999), representing 20–40% of total seed protein, and having a molecular weight in the range of 12–17 kDa (Stone et al., 2014; Von Der Haar et al., 2014). Their isoelectric point varies based on the method of extraction and the specific characteristics of the isoforms that exist (Stone et al., 2014). They are composed of two polypeptide chains, a 4.5 kDa small subunit and a large 10 kDa subunit, stabilized primarily by disulphide bonds. Their secondary structure shows a high α-helical content (Wu and Muir, 2008; Stone et al., 2014). Sequence comparison analyses have revealed that napin-type proteins share structural similarity with the prolamin superfamily of proteins which includes major allergens, α-amylase and trypsin inhibitors, and natural anti-fungal proteins (Breiteneder and Radauer, 2004).

Other proteins found in the seed are the oil body proteins including oleosins (which make up 6–8% of seed protein and 3% of the total oleosome weight) (Deleu et al., 2010; Von Der Haar et al., 2014), caleosins, steroleosins, and lipid transfer proteins (Terras F. R. et al., 1992), as well as myrosinase (thioglucoside glucohydrolase, the glucosinolate-degrading plant defense enzyme) (Gupta and Shaw, 2009; Gupta, 2010; Robin et al., 2017), Ca^+2^-dependent-calmodulin binding proteins, dehydrins (Wanasundara, 2011), and thionines, which have roles in plant defense (Jyothi et al., 2007; Pacheco-Cano et al., 2018).

Seeds of rapeseed (*Brassica napus* L.), black mustard (*B. nigra* Koch.), mustard collard (*B. carinata* A. Braun), white mustard (also called yellow mustard or Semen sinapis Albae) (*Sinapis alba*) have long been utilized in traditional medicine for relieving pain and infection, dyspnea, reducing nodulation, and relieving cough by eliminating phlegm, and as a tonic for stiffness, or muscle aching (Liu et al., 2003; Aboelsoud, 2010; Kabir et al., 2016; Khan et al., 2016; Rahman et al., 2018b). Mustard seeds are also traditionally exploited to prevent microbial growth of food-spoiling bacteria and increase the shelf life of processed food (Mir et al., 2017; Rahman et al., 2018b; Torrijos et al., 2019).

Evidence has suggested that 2S albumins may function in plant defense against pathogenic micro-organisms (Terras F. R. et al., 1992). Purified radish seed 2S albumins were shown to inhibit the growth of fungi and bacteria and together with thionines obtained both from wheat and barley origin increased cell wall permeabilization of the phytopathogens (Terras F. et al., 1992; Terras F. R. et al., 1992; Terras et al., 1993; Terras et al., 1996). Napin-like proteins from seeds of dwarf Chinese white cabbage (*Brassica rapa* L. syn. *Brassica chinensis* L.), and Chinese kale (*Brassica oleracea* L. syn. *Brassica alboglabra* cv. “Swatow”) both manifested antibacterial activity (Ngai and Ng, 2004). Homologs of 2S albumins from the seeds of other species with moderate to high amino acid sequence identity to rapeseed 2S albumin (Yang et al., 2007) were also reported to possess antimicrobial activities, for example, *Wrightia tinctoria* (Roxb.) R. Br. from Apocynaceae (Sharma et al., 2017), sesame, *Sesamum indicum* L. from Pedaliaceae (Maria-Neto et al., 2011) and castor bean, *Ricinus communis* L. from Euphorbiaceae (Yang et al., 2007). Protein rich defatted seed meal from *B. rapa* L. var. rapa DC, low in phytic acid and sinapine, demonstrated a broad-spectrum antimicrobial activity against food-borne pathogens (Tenore et al., 2012); however, it was not known what was the active protein/metabolite responsible for the activity, as glucosinolates have also been reported to have antimicrobial activity, and these compounds are found in high concentration in the seed meal along with SSP (Borges et al., 2015).

There is also some evidence that cruciferin proteins have anti-microbial activity. An 11S seed storage protein (SSP) from *Momordica cochinchinensis* (Lour.) Spreng., with significant amino acid sequence similarity to rapeseed cruciferins was thought to possess antimicrobial activity (Mazzio et al., 2018).

These findings support the hypothesis that the major mustard and rapeseed (*Brassica napus*, *B. juncea*, *B. nigra*, *B. rapa*, and *Sinapis alba*) SSPs, napin, and cruciferin could have antibacterial and antifungal activities based on their high sequence similarity to proteins from other species (Rahman et al., 2020). Although the seeds, seed paste and extracts of mustards were traditionally utilized and therefore extensively studied and reported for antimicrobial activities, there is no study on screening such biological activity using purified proteins despite their high sequence similarity to other confirmed antimicrobial proteins. Therefore, this article aims to determine the *in silico* and *in vitro* bioactivity of the rapeseed napin and cruciferin proteins to confirm their antimicrobial properties.

## Materials and Methods

### Amino Acid Sequence Analysis

Amino acid sequences of the major rapeseed 2S albumin and 12S globulin proteins including *B. napus*, *B. juncea*, *B. rapa*, and *S. alba*, and their corresponding peptides with reported antimicrobial activity (**Supplementary Table 1**), were collected from the Uniprot database (https://www.uniprot.org/, 12.11.19) and aligned using the program Clustal Omega (Madeira et al., 2019) and Mview (Madeira et al., 2019; Rahman et al., 2020). Sequence motifs related to potential antimicrobial function were identified (**Supplementary Table 1**) (Altschul et al., 1997; Schäffer et al., 2001). All published and publicly available protein sequences were selected based on gene ontology terms related to antimicrobial, antibacterial, and antifungal seed protein activity.

### SPRINT Database Search

To observe the protein family fingerprints, a group of conserved motifs used to characterize a protein family, SPRINT (http://130.88.97.239/dbbrowser/sprint/, 28.11.19) and its foundational interface PRINTS (http://130.88.97.239/PRINTS/index.php, 28.11.19) databases were searched using the protein name and amino acid sequences of the major rapeseed 2S albumin-napin and 12S globulin-cruciferin proteins (**Supplementary Table 1**) by using query “sequence” and “title” (Attwood et al., 2003).

### Antimicrobial Peptide Database Search

Four antimicrobial peptide databases were used to predict the antimicrobial activity of the rapeseed cruciferin and napin SSPs. These included two antimicrobial peptide databases, APD (http://aps.unmc.edu/AP/database/mysql.php), and AMPed (https://amped.uri.edu/index.php) (Wang et al., 2010; Wang et al., 2015), as well as the database of Antimicrobial Activity and Structure of Peptides (DBAASP) (https://dbaasp.org) (Pirtskhalava et al., 2015), and PhytAMP, a database dedicated to plant antimicrobial peptides (http://phytamp.pfba-lab-tun.org/main.php) (**Supplementary Table 1**).

### Three-Dimensional Structure Comparisons

Three-dimensional structure molecular modeling of the SSP was carried out using the program SWISS-MODEL (https://swissmodel.expasy.org/, 21.07.19) to compare structural similarity. High resolution three-dimensional theoretical structural models were generated. The 3D structural model of napin (P09893) was drawn using the structure of antibacterial sweet protein mabinlin-2 (PDB ID: 2DS2) as template. The 3D structural model of cruciferin was drawn based on the structure of antibacterial soybean glycinin (P04776, PDB ID: 1FXZ) from *Glycine max* (L.) Merr.

### *In Silico* Molecular Docking

In *in-silico* molecular docking studies of bio-active peptides or chemical drug molecules that exert their action by binding with specific receptors provides evidence on binding conformation, pattern and affinity. In this study, to determine the potential antimicrobial activity of napin and cruciferin proteins, their interaction with known bacterial receptors, including topoisomerase II (PDB ID: 1JIJ), DNA gyrase subunit b (PDB ID:1KZN) (Gullapelli et al., 2017), *Staphylococcus aureus* tyrosyl-tRNA synthetase (PDB id: 1JIJ), topoisomerase II DNA gyrase (PDB id: 2XCT) (Pisano et al., 2019), dihydrofolate reductase (PDB ID 3FYV), *Staphylococcus aureus* gyrase B (PDB ID 4URM), and *S. aureus* sortase A (PDB ID 2MLM) (Barakat et al., 2016), *S aureus* dihydrofolate reductase (PDB ID 3FRA) and the 50S ribosomal subunit from *Deinococcus radiodurans* (PDB ID: 1XBP) (Deng et al., 2019) were tested.

Three-dimensional structures of rapeseed (*B. napus*) 2S albumin napin (PDB ID: 1PNB) and procruciferin (PDB ID: 3KGL), as the closest available 12S globulin protein to cruciferin, and bacterial enzymes were obtained from the Research Collaboratory for Structural Bioinformatics Protein Data Bank (RCSB PDB) (http://www.rcsb.org/pdb, 26.12.19), the United States National Library of Medicine, National Center for Biotechnology Information server PubChem (https://pubchem.ncbi.nlm.nih.gov/, 26.12.19) and Royal Society of Chemistry chemical identifier search database (http://www.chemspider.com/Default.aspx, 26.12.19). Prior to analysis, water molecules (shown as 000, in the software) and other unwanted residues (recognized by characteristic sequence breaks) were removed from all proteins, when necessary, using PyMol (PyMOL™ v2.3.2 - Incentive Product, Schrodinger, LLC). The sequences were then subjected to energy minimization by Swiss-PdbViewer v4.1.0. The rapeseed proteins were then docked as protein ligands to the bacterial enzymes as receptors using PatchDock online docking server (https://bioinfo3d.cs.tau.ac.il/PatchDock). Results were refined and rescored utilizing Firedock (http://bioinfo3d.cs.tau.ac.il/FireDock/php.php, 26.12.19) which provides the global energy for the docked complexes (Duhovny et al., 2002; Schneidman-Duhovny et al., 2005; Andrusier et al., 2007). Usually, ligand-receptor binding energies are calculated using low-energy minima and compared with experimental values. The lowest binding energy/global energy in the solutions table was selected and the polar hydrogens were then added to the models using Biovia Discovery Studio 4.5 64-bit client (Jia et al., 2015; Oferkin et al., 2015).

### Amino Acid Composition

Earlier reports on antimicrobial peptides indicated that positively charged, glutamine-rich stretches of the peptide had the potential to aggregate bacterial cells, while bactericidal activity of the peptide involved hydrophobic proline residues within the protruding loop of the peptide (Suarez et al., 2005). Therefore, the amino acid composition of antibacterial napin proteins (P84529) from *Brassica rapa* subsp. chinensis (Pak-choi) (*Brassica chinensis*); napin (Allergen Sin a 1, P15322) from *Sinapis alba*, napin 2SSI_BRANA (P24565) from *B. napus*, and napin (Allergen Bra j 1-E, P80207) from *Brassica juncea*; and cruciferins (P33525, Cruciferin BnC2, P33524) from *B. napus* and cruciferin (P83908) from *Sinapis alba* were analyzed using ProtParam tool (https://web.expasy.org/protparam/, 26.12.19).

### Protein Characterization by 1D-SDS-PAGE and Western Blot Analysis

To confirm the purity of the napin and cruciferin proteins, 1D-SDS-PAGE was carried out according to Barkla et al. (2016). Protein was solubilized in SDS buffer (2% SDS) and loaded onto Mini-Protean^®^ TGX™ precast gels (7.2 cm × 8.6 cm gels, 1.0-mm thick, 15 well), and run using Tris/Glycine buffer system (BioRad USA). Gels were stained with Coomassie Blue stain and imaged using a Gel Doc XR imaging system, (Bio-Rad) with Image Lab Software (v.6.0.1).

To validate the presence of 2S albumin-type napins and cruciferins in the protein extracts separated by 1D-SDS-PAGE and confirm the purity, Western blotting was performed according to Barkla et al. (1999). Following SDS-PAGE, proteins were transferred electrophoretically onto prewetted polyvinylidene difluoride (PVDF) membranes (Bio-Rad) using a Trans-Blot^®^ Turbo™ Transfer System (Bio-Rad) at 2.5A, 25 V, 3 min in Turbo mode.

After transferring, the membrane was rapidly stained with Ponceau S stain [1% Ponceau S (w/v) in 5% acetic acid] on an orbital shaker for 1 min and then washed with MilliQ water to ensure correct transfer of proteins. Membranes were then blocked with 5% skim milk in TBS solution for 2 h on an orbital shaker and then incubated in blocking solution containing primary antibody overnight at room temperature. Primary antibodies against either cruciferin or napin (Shimada et al., 2003a; Shimada et al., 2003b) were used at 1/50,000 and 1/10,000 dilutions, respectively. After incubation with primary antibody, blots were washed 3 times (TBS, TBS + 0.1% tween 20, TBS) for 15 min and then incubated in goat anti-rabbit IgG (H&L) HRP conjugated secondary antibody (WesternSure HRP Goat Anti-Rabbit IgG - LiCOR^®^, USA). Chemiluminescent detection was performed using the WesternSure Chemiluminence kit (LiCOR^®^, USA) according to manufacturer’s specifications. The membrane was then scanned and digitized using a LiCOR^®^ C-Digit scanner (LiCOR) coupled with Image Studio v. 4 software.

### Antibacterial Activity Screening

#### Solubilization of Proteins

To observe antimicrobial activity, purified napin and cruciferin proteins from *B. napus* were purchased from Pilot Pflanzenöltechnologie Magdeburg e.V. (PPM), Magdeburg, Germany. Purified proteins were solubilized according to the standard method of handling and storing peptides (Turner et al., 2011). For disc diffusion and microdilution antimicrobial susceptibility testing (AST), 2 mg or 2.56 mg napin were each dissolved in 1-ml DNAse/RNAse free water. Napin dilutions were prepared in DNAse/RNAse free water or cation-adjusted Mueller-Hinton broth (CAMHB) to give 40, 20, 10, 5, 2.5, and 1.25 µg/20 µl for the disc diffusion AST, and 128, 64, 32, 16, 8, 4, 2, 1, 0.5, and 0.25 μg/ml for the microdilution AST, respectively. Two mg of water insoluble cruciferin (Rehder et al., 2017), was resuspended in 1 ml DNAse RNAse free water, 10% v/v acetic acid, and 20% v/v acetonitrile, to completely dissolve the protein. Cruciferin dilutions were prepared in DNAse/RNAse free water to give 30.8, 15.4, 7.7, 3.85, 1.925, and 0.9625 µg/20 µl for the disc diffusion AST. For microdilution AST, 2.56-mg cruciferin was dissolved in 1-ml dimethyl sulfoxide (DMSO), and dilutions were prepared in CAMHB to give 64, 32, 16, 8, 4, 2, 1, 0.5, 0.25, and 0.125 μg/ml and a final concentration of 1% DMSO.

#### Bacterial Cultures

Bacterial cultures from the American Type Culture Collection (ATCC) of both Gram-positive and Gram-negative bacteria, listed in **Table 1**, were prepared from pure cultures in Physical Containment 2 (PC2) facilities at the School of Health and Human Sciences, Southern Cross University, Australia. The bacteria were recovered from the −80°C microbank glycerol stocks, by reviving on Tryptic Soy agar or Columbia horse blood agar for 24 hours in a humidified incubator at 37°C, with 5% CO_2_.

**Table 1:** List of bacteria and controls used in the antimicrobial susceptibility tests.

Bacteria	Microdilution Antibiotic Positive Controls (ug/ml)	Microdilution Negative Controls
Gram negative bacteria		Blank: 100 μl CAMHB Growth control: 50 μl CAMHB: 50 μl inoculum 1% DMSO growth control: 1 μl DMSO in 50 μl CAMHB: 50 μl inoculum 2% DMSO growth control: 2 μl DMSO in 50 μl CAMHB: 50 μl inoculum Solvent growth control: 8.7 μl acetic acid: acetonitrile: water (1:2:7) in 50 μl CAMHB: 50 μl inoculum
*Enterobacter cloacae*	Ampicillin 128, 64, 32, 16, 8, 4, 2, 1, 0.5, and 0.25	
*Klebsiella oxytoca*		
*Escherichia coli*	Ampicillin 32, 16, 8, 4, 2, 1, 0.5, 0.25, 0.125, and 0.0625	
*Salmonella typhimurium*		
*Pseudomonas aeruginosa*	Gentamicin 32, 16, 8, 4, 2, 1, 0.5, 0.25, 0.125, and 0.0625	
Gram positive bacteria		
*Enterococcus faecalis*	Ampicillin 32, 16, 8, 4, 2, 1, 0.5, 0.25, 0.125, and 0.0625	
*Staphylococcus saprophyticus*	Ampicillin 4, 2, 1, 0.5, 0.25, 0.125, 0.0625, 0.031, 0.016, and 0.008	
*Staphylococcus aureus*		
*Streptococcus pyogenes*		

CAMHB, Cation-Adjusted Mueller-Hinton broth.

#### *In Vitro* Antimicrobial Activity Screening

Antimicrobial activity of the proteins was evaluated against four Gram positive and five Gram negative bacteria using two screening approaches. The disc diffusion AST as per the European Committee on Antimicrobial Susceptibility Testing (EUCAST) guidelines for measuring antimicrobial activity of proteins, and the microdilution AST as per the Clinical and Laboratory Standards Institute (CLSI) guidelines (Gullapelli et al., 2017).

All bacteria were prepared to visually match the turbidity of the 0.5 McFarland standard; giving a bacterial suspension of approximately 1 × 10^8^ CFU/ml. Briefly, small portions of four single, well isolated colonies from a pure culture (a dozen colonies were required for smaller colonial forms of *S. pyogenes*) were added to 4 ml sterile PBS, and mixed well to give a homogenous suspension. For the disc diffusion AST, four Mueller Hinton (MH) (Blood MH agar for *Streptococcus*) agar spread plates were prepared for each bacterium by evenly spreading 200 μl of the bacterial suspension (1 × 10^8^ CFU/ml) across the agar surface to create confluent growth.

Proteins at varying concentrations were tested against all bacteria shown in **Table 1**. Twenty μl of napin or cruciferin at the following concentrations (40, 20, 10, 5, 2.5, and 1.25 µg or 30.8, 15.4, 7.7, 3.85, 1.925, and 0.9625 µg, respectively) were added to six sterile filter paper discs, across two spread plates per bacteria. Twenty µl of water for the negative control was added to the centre blank disc on the spread plates testing napin, while 20 µl of the water/acetic acid/acetonitrile solvent for the negative control was added to the centre blank disc on the spread plates testing cruciferin. The antibiotic positive control discs impregnated with 5µg Ciprofloxacin (for *P. aeruginosa*) and 1.25 µg/23.75µg of Trimethoprim/Sulfamethoxazole (for all other bacteria) were placed in the quadrant for the positive control.

All plates were incubated for 18 hours in a humidified incubator at 37°C with 5% CO_2_. Following the incubation period, plates were observed for measurement of zones of inhibition and to record antimicrobial activity. Plate images are labeled digitally to reflect the actual concentrations tested.

All microdilution assays were performed in duplicate, and repeated once. A sterile 96 well plate was prepared for each bacteria by adding in duplicate, 50 μl antibiotic, napin, cruciferin and controls (100 μl CAMHB for blank), and 50 μl of a 1:100 CAMHB dilution of the bacterial suspension, giving a final concentration of 5 x 10^5^ CFU/ml in each well (see **Table 1** for bacteria, antibiotic concentrations, and controls). For *S. pyogenes* AST, 2.5 μl 50% lysed horse blood was added to all wells. The final concentrations of napin and cruciferin were 128, 64, 32, 16, 8, 4, 2, 1, 0.5, and 0.25 μg/ml, and 64, 32, 16, 8, 4, 2, 1, 0.5, 0.25, and 0.125 μg/ml, respectively.

All microdilution AST plates were sealed with parafilm and incubated for 16 to 20 hours (20 to 24 hours for *S. pyogenes*) in a humidified incubator at 37°C with 5% CO_2_. Following the incubation period, absorbance was measured at OD600 on the BioRad iMark Microplate Absorbance Reader to determine growth inhibition. The antimicrobial effect of napin and cruciferin against each bacteria was determined by observing a reduction in the absorbance measures, and their minimum inhibitory concentrations (MIC) were determined by comparing their absorbance to the blank absorbance. For any napin or cruciferin MIC observed, 10 μl of the bacterial suspension in the corresponding concentration, and in the two consecutive concentrations, was plated onto Blood MH agar, to determine the minimum bactericidal concentration (MBC).

## Results

### Amino Acid Sequence Analysis

Multiple sequence alignment of the major rapeseed 2S albumin and 12S globulin proteins (**Supplementary Table 1**), and their corresponding peptides and polypeptides revealed high sequence identity and conserved sequence motifs to a series of antimicrobial proteins reported from various plant species (**Supplementary Figures 1A, B**).

### SPRINT Database Search Results

**Figure 1:**
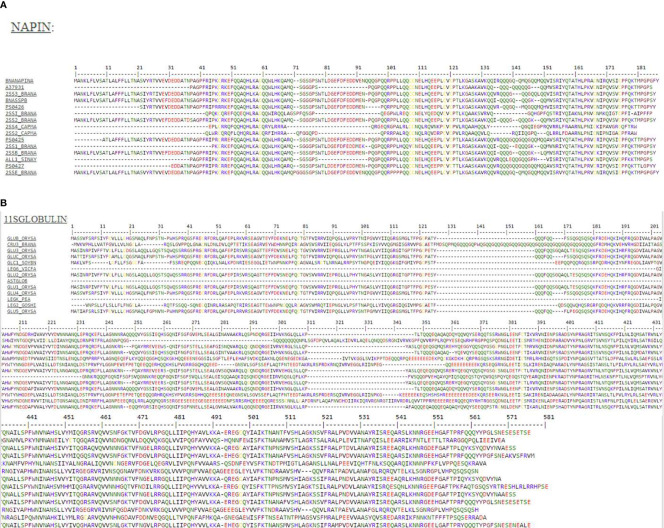
**(A)** Protein motif analysis of antimicrobial signatures (shown in the same color) in napin genes using the PRINTS database. BNANAPINA = Q7DMU4_BRACM = Napin (Q7DMU4) from *Brassica campestris*; 2SS1_BRANA = Napin-1 (P01091), 2SSI_BRANA = Napin-1A (P24565), 2SSB_BRANA = Napin-B (P27740), 2SS2_BRANA = Napin-2 (P01090), 2SS3_BRANA = Napin-3 (P80208) and 2SSE_BRANA = Napin embryo-specific protein (P09893) are from *Brassica napus*, ALL1_SINAL = Allergen Sin a 1 (P09893) is from *Sinapis alba*, and 2SS2_CAPMA = Sweet protein mabinlin-2 (P30233) and 2SS4_CAPMA =Sweet protein mabinlin-4 (P80353) are from *Capparis masaikai*. The values in the parenthesis are the Uniprot accession numbers (**Supplementary Table 1**). **(B)** Protein motif analysis of antimicrobial signatures (shown in the same color) in cruciferin genes using the PRINTS database. CRU3_BRANA = Cruciferin CRU1 (P33525) obtained from *Brassica napus*, GLUA1_ORYSJ = Glutelin type-A 1 (P07728), GLUA2_ORYSJ = Glutelin type-A 2 (P07730), GLUA3_ORYSJ = Glutelin type-A 3 (Q09151), GLUB1_ORYSJ = Glutelin type-B 1 (P14323) are obtained from *Oryza sativa subsp. japonica*; GLYG1_SOYBN = Glycinin G1 (P04776) from *Glycine max*, LEGA_GOSHI = Legumin A (P09802) from *Gossypium hirsutum*, LEGB6_VICFA = Legumin type B (P16079) from *Vicia faba*, LEGK_PEA = Legumin K (P05693) from *Pisum sativum*. The values in the parenthesis are the Uniprot accession numbers (**Supplementary Table 1**).

To search anti-microbial sequence motifs within the cruciferin and napin sequences, SPRINT, a compendium of diagnostic protein family fingerprints, was searched to determine sequence similarities to known antimicrobial peptides (Attwood et al., 2003). The SPRINT and PRINTS motif analysis by “user sequence query” of napin and cruciferin protein sequences (**Table 2**) identified true positive (napin with antimicrobial protein Q7DMU4, P80353, P30233, and cruciferin with antimicrobial protein P14323, Q09151, Q02897, P09802, and P04776) similar sequence scans (**Table 2** and **Figure 1**) (Mcinnis, 1998; Ramlan et al., 2002). The results indicated that napins and cruciferins are classified with some known antibacterial peptides and they have similar antimicrobial sequence motifs within their corresponding sequences.

**Table 2:** SPRINT database search results for napin and cruciferin.

Protein name	Organism	Reference/Uniprot accession no.
**“Napin”**		
BNANAPINA	*Brassica rapa* L. (syn. *Brassica campestris*)	Q7DMU4 (Q7DMU4_BRACM) (Mcinnis, 1998)
Napin-3	*Brassica napus* L.	P80208 (2SS3_BRANA)
Napin-1A	*Brassica napus* L.	P24565 (2SSI_BRANA)
Napin-2	*Brassica napus* L.	P01090 (2SS2_BRANA)
Sweet protein mabinlin-4	*Capparis masaikai* H.Lév.	P80353 (2SS4_CAPMA)
Sweet protein mabinlin-2	*Capparis masaikai* H.Lév.	P30233 (2SS2_CAPMA)
Napin-1	*Brassica napus* L.	P01091 (2SS1_BRANA)
Napin-B	*Brassica napus* L.	P27740 (2SSB_BRANA)
Allergen Sin a 1	*Sinapis alba* L.	P15322 (ALL1_SINAL)
Napin embryo-specific	*Brassica napus* L.	P09893 (2SSE_BRANA)
**Cruciferin**		
Glutelin type-B 1	*Oryza sativa* subsp. *japonica*	P14323 (GLUB1_ORYSJ)
Cruciferin CRU1	*Brassica napus* L.	P33525 (CRU3_BRANA)
Glutelin type-A 3	*Oryza sativa* subsp*. japonica*	Q09151 (GLUA3_ORYSJ)
Glutelin type-B 2	*Oryza sativa* subsp*. japonica*	Q02897 (GLUB2_ORYSJ)
Glycinin G1	*Glycine max* (L.) Merr.	P04776 (GLYG1_SOYBN)
Legumin type B	*Vicia faba* L.	P16079 (LEGB6_VICFA)
Glutelin type-A 2	*Oryza sativa* subsp*. japonica*	P07730 (GLUA2_ORYSJ)
Glutelin type-A 1	*Oryza sativa* subsp. *japonica*	P07728 (GLUA1_ORYSJ)
Legumin K	*Pisum sativum* L.	P05693 (LEGK_PEA)
Legumin A	*Gossypium hirsutum* L.	P09802 (LEGA_GOSHI)
Glutelin type-B 4	*Oryza sativa* subsp. *japonica*	P14614 (GLUB4_ORYSJ)

**Graphical Abstract:**
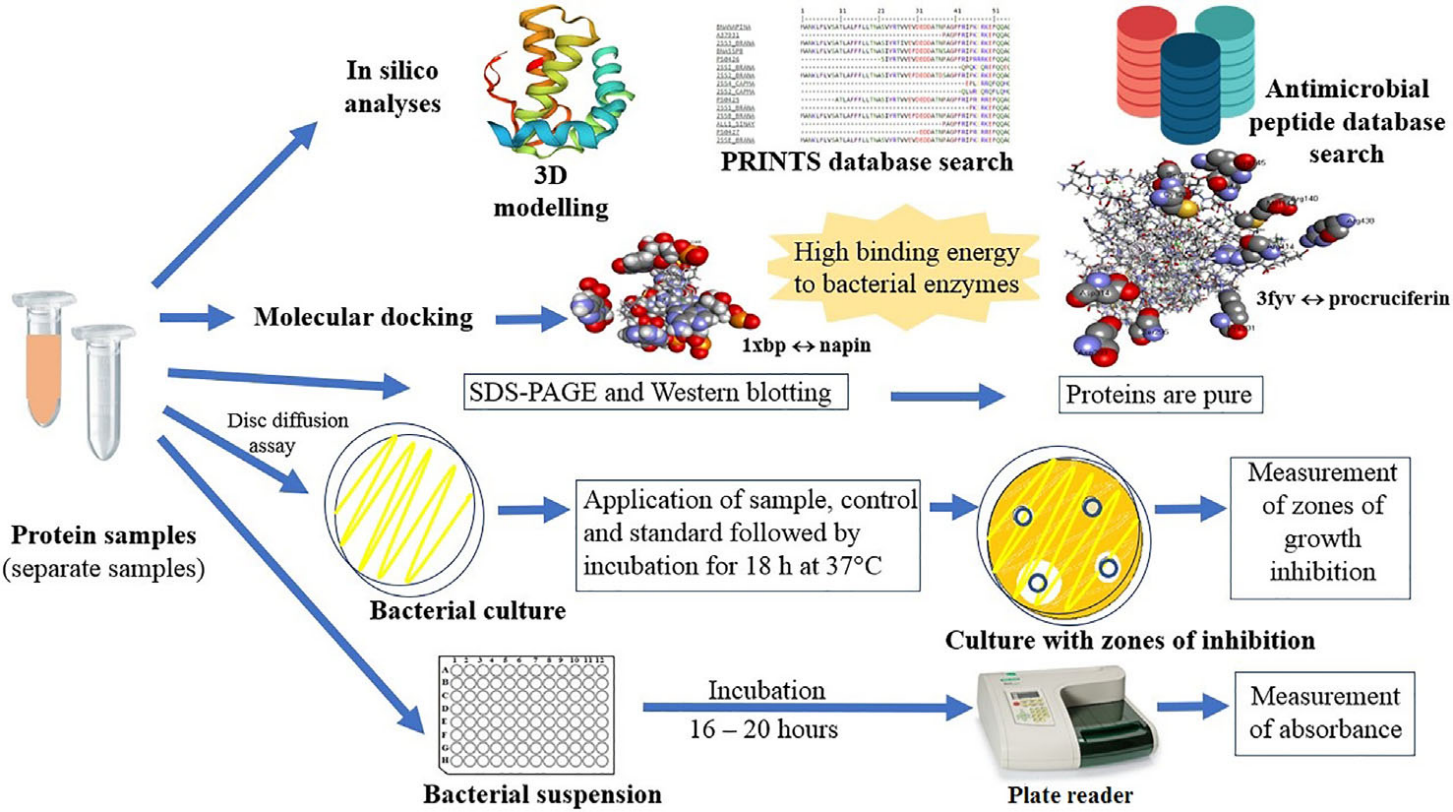


### Antimicrobial Peptide Database Search

One of the most direct methods to check if a query protein has been classified as an antimicrobial peptide based on experimental evidence is to carry out a search query in one of the antimicrobial peptide databases such as APD (http://aps.unmc.edu/AP/database/mysql.php), and AMPed (https://amped.uri.edu/index.php) (Wang et al., 2015). However, no hits in the target database were found for either napin or cruciferin proteins indicating that their antimicrobial activity has not been reported and/or included in the databases.

### Three-Dimensional Structure Comparisons

**Figure 2:**
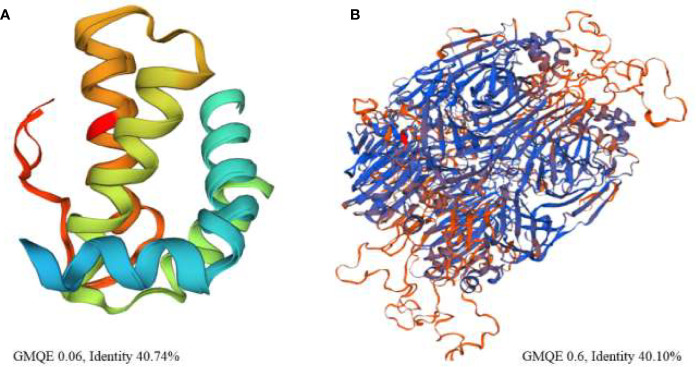
High resolution three-dimensional theoretical structural model of **(A)** napin from rapeseed (*Brassica napus*) (P09893) based on the structure of the antibacterial sweet protein mabinlin-2 (PDB ID: 2DS2) the template protein and **(B)** cruciferin based on the structure of antibacterial soybean glycinin (P04776, PDB ID: 1FXZ) from *Glycine max* the template protein. % identity is between the target protein and the template protein.

The three-dimensional structure of napin was found to have high homology (<40%) with mabinlin II (accession number P30233) from the seeds of *Capparis masaikai* H.Lev. which itself is an antimicrobial 2S albumin. Another antimicrobial polypeptide Flo (accession number P24303) from *Moringa oleifera* Lam. also shows homology to napin from *B. napus* (71% sequence identity) and mabinlin II, antimicrobial 2S SSPs from *Raphanus raphanistrum* subsp. *sativus* (L.) Domin (syn. *R. sativus* L.) and *Arabidopsis thaliana* (L.) Heynh. (Suarez et al., 2005). This implies that there is a high degree of similarity among flo, napin, mabinlin II, 2S SSPs from *R. sativus* and *A. thaliana* and several other proteins of the of 2S albumin SSP family from various plant species. High sequence similarity (>40%) was also observed between cruciferin and the soybean glycinin (PDB ID: 1OD5) (**Figure 2**) which has been reported to be a strong antimicrobial protein (Sitohy et al., 2012).

### *In Silico* Molecular Docking

The binding energy between the protein and the bacterial target receptor obtained from analysis of molecular docking supports a possible antibacterial role of the major rapeseed SSP. The global energy, atomic contact energy, attractive and repulsive short-range and high-range Coulomb electrostatics, PI-PI and cation-PI stacking, aliphatic interactions, and ligand transformation after refinement and residues involved in H-bonding are tabulated in **Tables 3A**–**C**.

**Table 3A:** Molecular docking scores of ligands (napin and cruciferin) and bacterial receptor proteins (1xbp, 3fra, 3fyv, and 4urm).

Complex name	Sol no	Global Energy	Softened attractive van der Waals energy	Softened repulsive van der Waals energy	Atomic contact energy	Insideness measure
**1xbp and napin**	7	−0.59	−1.61	0.49	−0.12	2.31
	5	9.18	0	0	0	13.12
	6	965.84	−41.1	1,273.52	3.16	5.16
	9	2,121.34	−24.46	2,707.98	−9.41	9.57
	1	5,698.16	−94.77	7,327.33	−16.89	7.82
	8	6,643.37	−89.32	8,488.29	−11.47	7.23
	3	7,793.08	−87.84	9,920.56	−12.14	11.15
	2	8,236.15	−62.31	1,0423.07	−7.39	4.25
	10	95,88.56	−126.96	1,2261.58	−19.94	2.33
	4	12,417.14	−133.42	1,5860.67	−47.34	6.38
**1xbp and procruciferin**	6	1.21	−8.06	2.94	−2.65	21.69
	10	8.12	−1.9	0.67	0.4	14
	7	10.63	−5.51	5.89	0.78	18.47
	8	11.63	−1.92	0.78	1.92	15.45
	4	12.24	−0.37	0	0.47	17.22
	5	931.29	−24.03	1,187.66	3.16	17.37
	9	2,271.55	−24.4	2,845.58	13.27	14.93
	1	2,348.57	−37.17	3,007.55	−9.82	20
	3	5,195.91	−48.03	6,616.99	−21.11	11.64
	2	6,642.51	−46.95	8,399.35	−16.86	29.19
3FRA and napin	3	9.67	−5.15	0.25	4.36	12.98
	7	11.99	−18.76	6.61	15.45	18.5
	6	13.94	−29.11	26.56	9.82	15.85
	2	19.23	−0.75	0	2.22	21.28
	10	356.25	−26.03	468.74	−3.02	3.91
	9	368.1	−38.18	481.56	12.76	13.88
	5	437.39	−38.47	571.12	7.64	9.96
	1	447.54	−29.03	593.26	0.16	5.97
	8	2,125.92	−58.88	2707.8	6.13	13.04
	4	2,921.04	−67.41	3751.22	7.48	12.65
3FRA and procruciferin	3	−18.4	−33.53	9.14	6.63	18.39
	9	7.07	−33.19	18.05	5.11	18.86
	8	12.74	−24.81	11.45	10.97	18.18
	10	13.78	−26.79	8.26	10.54	15.28
	1	14.38	−28.27	14.14	17.54	20.42
	7	24.06	−20.77	10.71	12.52	8.85
	5	31.73	−16.53	7.55	9.77	9.91
	6	35.27	−44.71	14.99	22.79	26.12
	4	46.04	−9.72	6.78	8.46	20.11
	2	1,413.49	−39.03	1752.69	13.97	16.75
3fyv and napin	7	10.1	−2.74	0.6	3.02	19.22
	10	28.13	−23.99	12.28	12.2	15.36
	8	37.82	−34.61	28.57	12.7	18.12
	2	39.93	−29.2	26.67	18.88	15.78
	3	45.64	−18.73	17.01	13.08	16.78
	9	116.89	−23.22	118.17	18.87	16.1
	6	956.15	−39.5	1,267.32	0.69	9.89
	5	1,428.16	−22.18	1,797.91	14.11	11.47
	1	2,298.62	−51.15	2,982.34	−1.48	10.44
	4	4,226.41	−53.66	5,383.38	3.23	11.43
3fyv and procrucifrerin	3	−18.35	−31	19.02	6.1	18.91
	1	−12.47	−36.55	20.82	6.23	20.75
	2	−7.57	−20.79	17.3	6.92	16.75
	8	−1.86	−18.25	4.4	4.97	15.95
	7	6.68	−19.44	19.24	8.98	13.41
	10	12.21	−23.53	23.31	11.27	17.97
	5	35.64	−22.45	10.36	11.19	21.6
	9	39.63	−24.11	8.84	15.88	20
	6	684.27	−43.27	942.18	1.59	15.88
4urm and napin	7	−9.16	−23.05	4.03	13.03	10.4
	6	−6.66	−7.2	0.69	0.73	3.46
	8	−3.81	−25.16	11.33	15.72	17.27
	5	−3.23	−14.12	3.96	5.69	8.62
	2	1	−3.22	0	1.48	11.06
	10	6.79	−7.07	0.22	7.88	9.47
	3	12.67	−1.61	0	1.56	16.15
	9	14.97	−18.85	13.87	14.34	12.75
	1	58.84	−12.38	36.52	5.95	7.38
	4	317.09	−46.6	421.44	16.89	10.47
4urm and procruciferin	10	2.83	−2.08	0	3.29	10.25
	6	6	−8.97	3.59	1.89	16.23
	2	6.62	−8.84	6.65	−0.29	19.8
	4	11.86	−31.63	10.19	15.62	16.63
	5	20.54	−4.71	0.12	−0.24	22.41
	3	34.18	−7.81	0.91	5.68	16.7
	9	42.57	−18.89	2.96	13.63	16.95
	1	55.59	−32.57	24.05	7.35	21.79
	8	85.15	−27.61	93	13.49	20.4
	7	1,252.18	−18.68	1,600.34	−0.04	6.92

Solution numbers are arranged according to the order of the input transformations or models by default settings of online docking server. Global Energy is the binding energy of the solution complex. aVdW, rVdW, softened attractive and repulsive van der Waals energy; ACE, atomic contact energy; inside, insideness measure; aElec, rElec, attractive and repulsive short-range Coulomb electrostatics; laElec, lrElec, attractive and repulsive long-range Coulomb electrostatics; HB, hydrogen and disulfide bonds; CHB, Conventional Hydrogen Bond; piS, PI-PI stacking; catpiS, cation-PI stacking; aliph, aliphatic interactions; Transformation, ligand transformation after refinement. Analysis of the binding interactions of napin and procruciferin with bacterial enzymes showed that the ligand (napin and procruciferin) is surrounded by the amino acids of the bacterial enzymes mentioned in column H. Elec, Electrostatic; AC, Attractive Charge. 1XBP is the 50S ribosomal subunit from Deinococcus radiodurans, 3FRA is Staphylococcus aureus dihydrofolate reductase, 4URM is S. aureus gyrase B, and 3FYV is S. aureus dihydrofolate reductase. Bacterial receptor proteins were selected by efficient global search of relative receptor-ligand interactions (Andrusier et al., 2007; Barakat et al., 2016).

**Table 3B:** Molecular docking scores of ligands (napin and cruciferin) and bacterial receptor proteins (1xbp, 3fra, 3fyv, and 4urm).

Complex name	Sol no	aElec	rElec	laElec	lrElec	HB	piS	catpiS	aliph	Transformation
**1xbp and napin**	7	0	0	0	0	0	0	0	0	−2.182 0.785 −0.167 153.825 85.611 72.471
	5	0	0	0	0	0	0	0	0	−2.546 0.068 0.002 107.452 28.385 110.538
	6	0	0	0	0	0	0	0	0	3.055 0.849 −1.166 90.032 169.446 145.327
	9	0	0	0	0	0	0	0	0	2.831 0.483 −1.078 114.903 66.700 61.513
	1	0	0	0	0	0	0	0	0	−2.716 −0.099 −0.070 103.328 74.379 76.142
	8	0	0	0	0	0	0	0	0	−0.435 −0.617 1.439 100.459 74.442 79.054
	3	0	0	0	0	0	0	0	0	−0.432 0.132 −3.035 101.192 71.071 75.851
	2	0	0	0	0	0	0	0	0	−0.446 0.219 −2.705 102.389 70.811 74.034
	10	0	0	0	0	0	0	0	0	−1.959 −0.076 −2.320 100.384 71.509 77.724
	4	0	0	0	0	0	0	0	0	2.622 −0.218 −1.907 109.921 69.589 72.565
**1xbp and procruciferin**	6	0	0	0	0	0	0	0	0	−1.479 −0.555 −0.584 157.666 116.809 83.178
	10	0	0	0	0	0	0	0	0	0.050 −0.527 −0.712 157.651 121.770 178.506
	7	0	0	0	0	0	0	0	0	0.861 −0.884 −2.532 132.500 −12.579 49.529
	8	0	0	0	0	0	0	0	0	−2.937 0.836 −2.558 120.442 27.038 99.815
	4	0	0	0	0	0	0	0	0	0.305 0.080 2.319 93.522 27.314 125.913
	5	0	0	0	0	0	0	0	0	−2.781 −0.979 −1.829 124.149 −5.545 92.508
	9	0	0	0	0	0	0	0	0	−0.348 −0.341 1.407 135.052 73.380 18.199
	1	0	0	0	0	0	0	0	0	−2.786 −0.160 −2.122 86.345 −2.855 45.882
	3	0	0	0	0	0	0	0	0	0.014 −0.912 2.859 36.535 187.629 −33.873
	2	0	0	0	0	0	0	0	0	−2.169 −0.881 2.534 136.756 46.109 89.104
**3FRA and napin**	3	0	0	0	3.65	−1.16	0	0	0	−0.374 0.333 −2.558 48.513 22.971 58.415
	7	0	2.53	0	5.59	−4.27	0	−3	0	2.498 0.263 0.565 31.811 −9.349 22.582
	6	−39.57	49.91	−14.84	25.75	−3.61	−5	−1.5	0	0.777 −0.920 0.031 31.240 −6.76720.236
	2	−18	13.15	−5.6	4.24	0	0	0	0	−1.844 −0.378 2.308 33.992 13.213 72.834
	10	0	44.64	0	28.65	−3.13	−3	0	0	−1.866 0.052 −1.199 54.995 6.205 39.585
	9	−31.84	52.4	−2.64	20	−5.53	−1.5	0	0	2.809 −0.276 −1.675 32.913 −6.274 20.409
	5	−3.67	92.55	−8.86	14.77	−5.3	−3.5	0	0	−1.765 −0.023 −1.244 52.431 5.865 40.971
	1	−4.3	12.91	−3.53	27.28	−4.66	−3	0	0	−1.910 −0.035 −1.076 55.277 9.872 38.750
	8	0	168.99	−13.9	16.5	−8.96	−6	−1.5	0	1.154 −0.855 0.068 30.797 −3.343 19.271
	4	−18.94	63.43	−5.72	13.39	−7.83	−8.5	−0.5	0	−2.319 −0.115 −2.030 42.600 −6.846 29.913
**3FRA and procruciferin**	7	−63.76	76.16	−26.94	7.65	−2.6	−0.5	−4.5	0	−2.395 −0.295 3.104 −40.270 5.564 75.524
	5	−47.32	155.69	−20.65	12.73	−7.49	−1.5	0	0	−2.400 1.033 1.077 61.649 63.589 28.921
	6	−33.43	83.8	−15.12	5.66	−2.01	−0.5	−1.5	0	−1.829 −1.362 −0.374 −21.860 23.311 19.302
	9	−9.05	70.71	−19.39	24.64	−4.39	−3.5	−2	0	2.402 −0.332 −1.654 −27.722 −6.950 50.787
	1	−77.85	44.59	−31.15	25.54	−3.36	−3.5	0	0	−1.451 1.285 −1.855 53.382 −39.770 50.677
	8	−9.3	6.63	−11.71	35.54	−1.28	−2	0	0	−0.997 −0.228 0.402 67.781 63.930 47.546
	3	−35.06	88.26	−21.91	27.64	−1.53	−0.5	0	0	0.037 −0.982 2.915 −18.447 −36.805 −29.202
	2	−97.95	166.22	−19.3	37.74	−2.73	−1.5	0	0	−1.708 −0.533 −1.055 −17.817 −34.684 8.139
	10	0	97.58	−9.94	14.63	−2.21	0	−0.5	0	−1.396 −0.553 0.583 50.656 74.284 19.120
	4	−7.53	126.93	−26.95	30.3	−4.65	−4.65	−3	0	2.312 −0.378 −1.677 −26.461 −9.600 53.378
**3fyv and napin**	7	−18.13	0	−2.56	0	−0.63	0	0	0	1.195018 0.111900 −0.404503 36.603848 13.070419 67.113762
	10	−4.79	57.17	−8.34	26.33	−0.92	−3	−1.5	0	1.419516 1.422478 −0.341556 48.927547 4.558572 42.616772
	8	0	113.12	−2.71	24.28	−3.99	−1.5	−0.5	0	0.897423 −0.512778 0.087532 38.153831 −4.305618 22.148920
	2	−12.15	64.62	−5.29	25.39	−4.08	−2.5	0	0	0.571090 −0.669658 −0.104022 34.059589 −7.079601 22.993988
	3	0	33.55	−2.32	31.86	−1.32	0	0	0	0.327455 1.276970 2.856120 54.459126 8.206043 44.482044
	9	0	38.48	−2.61	14.07	−1.43	0	−0.5	0	2.365478 0.011664 1.090053 35.474125 −9.137678 22.458466
	6	−4.16	0	−7.4	3.81	−4.35	−3	0	0	0.186018 −0.592759 2.217820 21.126738 36.698421 53.015606
	5	−73.96	26.78	−27.29	−27.29	−4.61	−1.5	−1.5	0	−1.635767 −0.367702 1.973211 6.267393 32.485970 42.305016
	1	0	0	−2.41	0	−10.39	−3	0	0	0.183982 −0.287892 1.906260 25.036688 33.092117 58.026882
	4	−5.03	0	−8.11	0	−8.82	−1	−0.5	0	−0.185922 0.394067 −1.593941 26.694721 12.248711 70.375458
**3fyv and procrucifrerin**	3	−98.07	83.04	−17.98	9.03	−8.49	−0.5	−3.5	0	0.882312 0.270622 0.452004 34.376209 16.761656 105.629204
	1	−61.41	84.22	−22.21	5.66	−2.95	−1.5	−3.5	0	−2.408909 −0.310804 3.137617 −40.343742 3.093425 74.197830
	2	−140.35	79.66	−6.04	7.93	−3.92	0	−1.5	0	−0.871412 0.289663 −1.703906 −3.532524 −71.564064 81.714600
	8	−71.86	42.05	−18.43	25.3	−1.69	−1.5	−1.5	−1.5	−2.924418 −1.277309 −0.012886 −4.014475 −30.156733 16.353909
	7	−19.67	5.26	−2.47	4.59	−2.47	−0.5	0	0	−1.000771 −0.166307 2.180230 29.872753 95.077187 34.847866
	10	−4.48	2.47	−16.22	1.67	−0.83	−1.5	0	0	1.584941 1.442978 1.329863 17.483091 −43.860760 60.712036
	5	−44.84	84.56	−22.3	40.69	−4.93	−2.5	0	0	−2.497311 1.305315 −0.634158 63.377178 −31.635798 44.732738
	9	−31.71	130.52	−30.34	19.31	−2.67	−0.5	−1.5	0	−2.693943 0.193235 0.101170 114.073677 8.710386 87.306931
	6	−68.99	58.52	−39	11.46	−12.24	−5.5	0	−1.5	0.541106 0.042602 −0.569371 98.065758 −28.758331 7.812671
	3	0	90.92	−3.16	11.64	−7.58	−4.5	−0.5	−0.5	−0.486131 1.271754 −2.446670 46.617748 −29.963055 47.164417
**4urm and napin**	7	−98.45	64.66	−54.86	8.7	−2.99	0	0	−0.5	1.868066 −0.092131 1.525814 3.637999 −3.130833 89.582695
	6	−21.61	5.28	−8.68	9.4	−1.67	0	−1.5	0	−1.460179 0.502492 1.252873 −0.179193 −0.899414 90.955017
	8	−37.47	2.22	−18.01	6.93	−7.41	−0.5	−0.5	0	−2.274868 −0.086826 1.442803 −1.433655 −8.620634 21.917574
	5	0	0	−11.27	5.51	−3.48	0	0	0	2.159986 0.164253 −1.314865 0.505913 −3.224665 87.916992
	2	−17.01	0	−2.35	0	−0.59	0	0	0	−1.886352 −0.360272 2.477731 −30.973713 −4.360527 86.722488
	10	−3.7	0	−23.82	0	0	0	0	−0.5	−2.866194 0.273189 −1.994032 −2.115814 0.507240 85.711090
	3	0	0	0	3.38	−0.87	0	0	0	−0.317704 −0.848188 −0.778242 1.825974 0.354532 101.007179
	9	−77.7	42.28	−23.21	15.08	−2.23	0	0	0	1.982411 0.129197 −0.731296 3.800283 3.508109 88.668083
	1	−26.53	96.95	−11.3	33.03	−0.43	0	−1.5	−1	−2.776647 −0.022754 2.788598 −6.041031 −2.748431 83.736801
	4	−13.84	65.53	−22.31	18.81	−3.28	−3	−2	0	2.470320 0.397040 −2.668524 6.743612 −16.430897 23.362900
**4urm and procruciferin**	10	−36.17	5.34	−14.5	0	0	0	0	0	1.135997 −0.824196 −0.303326 0.146988 1.550484 90.916000
	6	−22.1	14.31	−4.85	8.27	−1.27	0	−0.5	0	0.572244 1.138489 1.520022 54.118980 70.514359 110.521210
	2	0	0	0	6.37	−1.25	−0.5	0	−0.5	0.100003 1.048633 −0.000666 96.403412 −40.060814 111.336456
	4	−65.72	98.02	−36.28	28.24	−2.67	0	−1.5	−3	−2.418812 0.821444 −1.755734 −42.335209 −13.986760 148.038712
	5	0	29.32	−3.1	8.75	0	0	0	0	2.891166 −0.650604 −1.210649 −9.728887 −14.524008 98.395508
	3	0	32.64	0	25.43	0	0	0	0	−1.590922 0.173499 −0.392852 10.746419 5.179658 91.331253
	9	−12.58	77.45	−24.05	37.72	−3.99	0	0	0	−2.217826 −1.256601 −2.375825 −26.934299 46.748051 64.341164
	1	−72.36	167.22	−25.94	63.32	−3.48	0	0	−0.5	−2.266007 0.966959 −2.883063 25.514585 −12.810349 162.878510
	8	0	52.53	−2.58	11.68	−2.33	0	0	0	1.868728 0.001705 −0.450134 102.784889 19.933956 61.477333
	7	0	0	0	0	−4.04	0	0	0	0.937499 0.348988 1.225773 9.959099 −7.660954 132.986313

aElec, rElec, attractive and repulsive short-range Coulomb electrostatics; laElec, lrElec, attractive and repulsive long-range Coulomb electrostatics; HB, hydrogen and disulfide bonds; piS, PI-PI stacking; catpiS, cation-PI stacking; aliph, aliphatic interactions; Transformation, ligand transformation after refinement; Sol no, Solution number. 1XBP is the 50S ribosomal subunit from Deinococcus radiodurans, 3FRA is Staphylococcus aureus dihydrofolate reductase, 4URM is S. aureus gyrase B, and 3FYV is S. aureus dihydrofolate reductase. All the other sequences were retrieved from the GenBank database and are as follows (source species and sequence accession numbers are shown in parenthesis): Mabinlin-II (Capparis masaikai; P30233), Sesa1 (Arabidopsis thaliana; P15457), Napin-2 (Brassica napus; P01090), Sin a 1 (Sinapis alba; P15322), Napin-1A (B. napus; P24565), Ric c 3 (Ricinus communis; P01089), Ber e 1 (Bertholletia excelsa; P04403), Ric c 1 (R. communis; P01089), and Gm2S-1 (Glycine max; P19594). Bacterial receptor proteins were selected by efficient global search of relative receptor-ligand interactions (Andrusier et al., 2007; Barakat et al., 2016).

**Table 3C:** Ligand interactions of the docked complexes with the least global energy of a category.

The name of the complex	Residue active site	Distance	Bond category	Type of H bond
1xbp and Napin (solution no. 7)	0:G1:H1 - 0:C2876:N3	1.58883	HB	CHB
	0:G1:H22 - 0:C2876:O2	1.38916	HB	CHB
	0:G2:H1 - 0:C2875:N3	1.97492	HB	CHB
	0:G2:H22 - 0:C2875:O2	1.43512	HB	CHB
	0:U3:H3 - 0:A2874:N1	1.50205	HB	CHB
	0:C4:H41 - 0:G2873:O6	2.94867	HB	CHB
	0:A5:H62 - 0:U2871:O4	2.9457	HB	CHB
	0:A5:H62 - 0:U2872:O4	2.48541	HB	CHB
	0:A6:H62 - 0:U2871:O4	2.42061	HB	CHB
	0:G7:H1 - 0:C2870:N3	1.64556	HB	CHB
1xbp and procruciferin (solution no. 6)	0:G2044:HO2’ - 0:C2046:OP2	2.9279	HB	CHB
	0:G2044:H1 - 0:A2430:N1	3.09807	HB	CHB
	0:G2044:H22 -0:MUL2881:O4	1.52911	HB	CHB
	0:A2430:H62 - 0:G2044:O6	2.63632	HB	CHB
	0:A2482:HO2’ - 0:A2482:O3’	1.76876	HB	CHB
	0:MUL2881:H1 - 0:A2482:N3	2.9486	HB	CHB
	0:G2044:H3’ - 0:G2044:OP2	2.75595	HB	CaHB
	0:G2044:H1’ - 0:A2482:N3	2.33872	HB	CaHB
	0:G2044:H8 - 0:A2482:O4’	2.86672	HB	CaHB
	0:A2045:H8 - 0:A2045:O5’	1.93278	HB	CaHB
3FRA and napin (solution no. 3)	A:LYS4:NZ - A:GLU8:OE2	5.04158	Elec	AC
	B:ARG11:NH1 - B:GLU12:OE2	5.06307	Elec	AC
	B:GLN6:HE22 - A:GLN1:OE1	2.31724	HB	CHB
	A:GLN6:HN - A:PRO2:O	2.41349	HB	CHB
	A:GLN6:HE21 - A:PRO2:O	3.01746	HB	CHB
	A:ARG7:HN - A:GLN3:O	1.64455	HB	CHB
	A:GLU8:HN - A:LYS4:O	1.55533	HB	CHB
	A:PHE9:HN - A:CYS5:O	1.92149	HB	CHB
	A:GLN10:HN - A:GLN6:O	2.24995	HB	CHB
	A:GLN10:HN - A:ARG7:O	2.43412	HB	CHB
3FRA and Procruciferin (solution no. 7)	X:THR1:HT2 – X:GLY87:O	2.3925	HB	CHB
	X:LEU2:HN – X:ASP106:OD2	1.62089	HB	CHB
	X:SER3:HN – X:VAL89:O	1.77077	HB	CHB
	X:SER3:HG – X:ASP107:OD2	1.58089	HB	CHB
	X:ILE4:HN – X:ASP107:O	1.99662	HB	CHB
	X:LEU5:HN – X:ILE91:O	1.76104	HB	CHB
	X:VAL6:HN – X:TYR109:O	2.05314	HB	CHB
	X:HIS8:HN – X:THR111:O	1.85144	HB	CHB
	X:HIS8:HD1 – X:ASP9:0	1.70772	HB	CHB
	X:ASP9:HN – X:VAL13:O	2.01965	HB	CHB
3fyv and napin	A:GLN1:HE22 - A:GLN3:OE1	2.92555	HB	CHB
	A:GLN6:HN - A:PRO2:O	2.44912	HB	CHB
	A:GLN6:HN - A:GLN3:O	2.95937	HB	CHB
	A:ARG7:HN - A:GLN3:O	1.64497	HB	CHB
	A:GLU8:HN - A:LYS4:O	1.72404	HB	CHB
	A:GLU8:HN - A:GLU8:OE1	2.77826	HB	CHB
	A:PHE9:HN - A:CYS5:O	1.85894	HB	CHB
	A:GLN10:HN - A:GLN6:O	2.24342	HB	CHB
	A:GLN10:HN - A:ARG7:O	2.32573	HB	CHB
	A:GLN11:HN - A:ARG7:O	1.73477	HB	CHB
3fyv and procrucifrerin	X:SER136:HG - A:THR86:O	3.0381	HB	CHB
	A:THR86:OG1 - X:GLU138:OE2	2.27006	HB	CHB
	B:ARG414:NH1 - X:LEU10:O	2.72974	HB	CHB
	X:SER136:HB2 - A:THR86:O	2.89323	HB	CHB
	X:THR1:HT2 - X:GLY87:O	2.30591	HB	CHB
	X:LEU2:HN - X:ASP106:OD2	1.86004	HB	CHB
	X:SER3:HN - X:VAL89:O	1.79673	HB	CHB
	X:SER3:HG - X:ASP107:OD2	1.62549	HB	CHB
	X:ILE4:HN - X:ASP107:O	2.0698	HB	CHB
	X:LEU5:HN - X:ILE91:O	1.78562	HB	CHB
4urm and napin	C:SER38:OG - D:GLU186:OE2	2.74016	HB	CHB
	C:SER40:OG - D:GLU186:OE1	2.76232	HB	CHB
	C:SER40:OG - D:GLU186:OE2	2.90773	HB	CHB
	D:SER40:HG - C:GLU186:OE2	3.03646	HB	CHB
	D:ARG42:HH22 - C:GLU186:O	1.6245	HB	CHB
	C:SER38:OG - D:GLU186:OE2	2.74016	HB	CHB
	C:SER40:OG - D:GLU186:OE1	2.76232	HB	CHB
	C:SER40:OG - D:GLU186:OE2	2.90773	HB	CHB
	D:ARG29:HH11 - D:ALA133:O	1.80548	HB	CHB
	D:LYS30:HN - D:GLU26:O	2.13732	HB	CHB
4urm and procruciferin	B:ILE183:HN - B:ASP180:O	2.37223	HB	CHB
	B:PHE184:HN - B:ASP180:O	1.85648	HB	CHB
	B:THR185:HN - B:ILE183:O	2.37084	HB	CHB
	B:THR187:HG1 - B:VAL189:O	2.97098	HB	CHB
	B:TYR190:HN - B:ASN75:OD1	2.23923	HB	CHB
	B:ASN191:HN - B:HIS45:NE2	1.84241	HB	CHB
	B:TYR192:HH - B:ASP225:OD1	2.1739	HB	CHB
	B:GLU193:HN - B:ASN191:OD1	2.81969	HB	CHB
	B:THR194:HN - B:ASN191:O	2.67423	HB	CHB
	B:THR194:HN - B:ASN191:OD1	2.43501	HB	CHB

**Figure 3:**
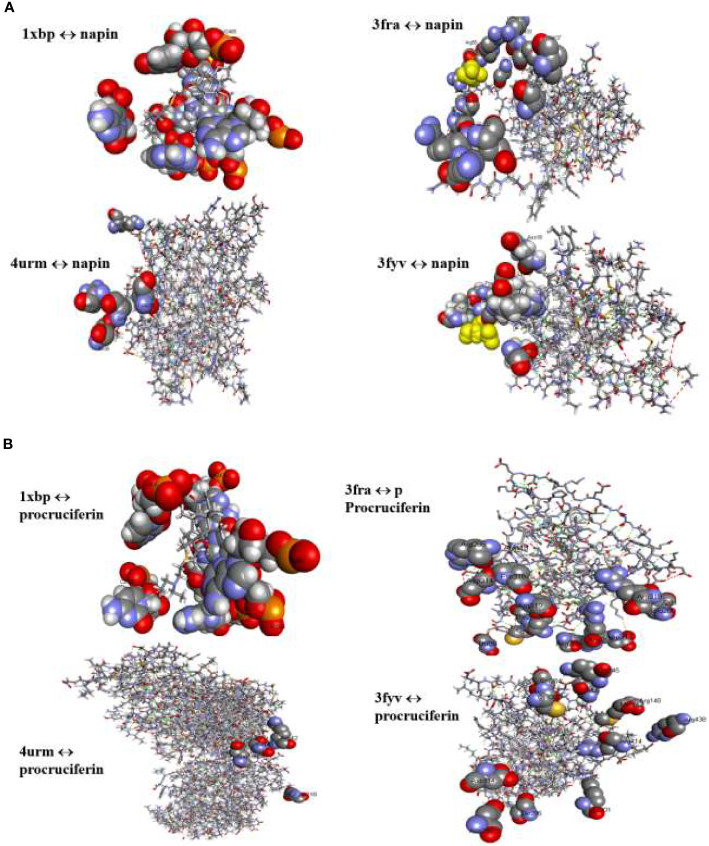
**(A)** Binding interactions of napin ligand with different proteins using Patchdock. 1XBP is the 50S ribosomal subunit from *Deinococcus radiodurans*, 3FRA is *Staphylococcus aureus* dihydrofolate reductase, 4URM is *S. aureus* gyrase B, and 3FYV is *S. aureus* dihydrofolate reductase. **(B)** Binding interactions of procruciferin with different proteins using Patchdock. 1XBP is the 50S ribosomal subunit from *Deinococcus radiodurans*, 3FRA is *Staphylococcus aureus* dihydrofolate reductase, 4URM is *S. aureus* gyrase B, and 3FYV is *S. aureus* dihydrofolate reductase.

Among the bacterial enzymes used in the docking analysis, four were found to interact with rapeseed proteins. The docking simulation of the rapeseed proteins with bacterial enzymes at the active site are presented in **Figures 3A, B**. Assessment of the top ten conformations were completed and the conformation having the lowest atomic energy value (Kcal/mol) was processed for post dock analysis using Biovia Discovery Studio 4.5 64-bit client. Hydrogen bond and non-bond interactions were then added (Riaz et al., 2019). Assessment of the two-dimensional design was undertaken to check the most extreme restricting interactions of the complex framed amongst amino acid residues and ligands.

Patch Dock provides several model solution options and, in all cases, the best “Solution” was nominated for each different single docking as the most optimal model as it surrounded the most critical residues for the binding pocket for docking analyses assigned to the crystal structure of the target receptor site (Batool et al., 2018). The binding affinities of the docked proteins (napin and procruciferin) were evaluated as scores and the Atomic Contact Energy (ACE) of the protein-receptor docked complexes were calculated. The hydrogen bonding and hydrophobic interactions of napin and procruciferin were measured within the attachment site of the receptor protein. The theoretical conformation of the ligands with the highest biological activity is presented in **Table 3** and **Figure 3** with favorable contacts with the attachment site presented. The docked structures were examined by using Discovery Studio 4.5 Visualizer (Jia et al., 2015; Rehman et al., 2018; Bai et al., 2019; Riaz et al., 2019) and Chimera 1.9 (Withana-Gamage et al., 2011; Nietzel et al., 2013; Withana Gamage, 2013).

The results indicate that both napin and cruciferin proteins have high binding affinity to selective bacterial enzymes, suggesting they have the potential to inhibit bacterial activity. As far as global energy is concerned, the lowest value describes the best binding energy (Aamir et al., 2018). Based on this, it can be conjectured that napin specifically binds better with the 50S ribosomal subunit from *Deinococcus radiodurans* (PDB ID: 1XBP) and *Staphylococcus aureus* gyrase B (PDB ID 4URM) than procruciferin. In contrast, procruciferin binds better with dihydrofolate reductase (PDB ID 3FYV) and *S. aureus* dihydrofolate reductase (PDB ID 3FRA) than napin. In addition to these observations, the overall binding performance of procruciferin is more intense than napin. The molecular docking study thus indicates procruciferin is a better antimicrobial agent than napin. Well established drug molecules Trimetrexate, Pyrimethamine, Methotrexate, and Trimethoprim were reported to bind with 3FRA (Schomburg, 2014). It is also evident from the results that both the napin and cruciferin proteins bind with the 50S ribosomal subunit from *Deinococcus radiodurans* (1XBP) through hydrogen bonding. Novel pleuromutilin derivative antibacterial compounds with substituted amino moieties were reported to exert antibacterial activity by binding to 1XBP (Shang et al., 2013).

### Amino Acid Composition

The amino acid composition analysis indicates that napin and cruciferin have positively charged, glutamine-rich stretches similar to other antimicrobial peptides that are involved in aggregating bacterial cells (**Table 4**) (Suarez et al., 2005). Additionally, bactericidal activity of the peptide involves hydrophobic proline residues within the protruding loop of the peptide (Suarez et al., 2005). The amino acid composition analysis of 2SSI_BRANA (P24565), Cruciferin Cru 1 (P33525) and Cruciferin BnC2 (P33524) are given in **Table 4**.

**Table 4:** Amino acid composition of napin and cruciferins.

	Napin, 2SSI_BRANA (P24565)		Cruciferin Cru 1 (P33525)						Cruciferin BnC2 (P33524)				
	Number of amino acids in napin	Total % of amino acids	Number of amino acids in cruciferin Signal (1–23)	Number of amino acids in alpha chain (24–319)	Number of amino acid in beta chain (320–509)	Total amino acid count	Total % of amino acids	Number of amino acids in cruciferin signal (1–23)		Number of amino acids in alpha chain (24–306)	Number of amino acids in beta chain (307–496)	Total amino acids count	Total % of amino acids	
Ala (A)	6	5.5	2	11	16	29	6.933	2		16	15	33	7.43	
Arg (R)	6	5.5	0	17	12	29	4	1		13	14	28	5.43	
Asn (N)	4	3.6	1	15	16	32	5.9333	0		17	12	29	4.1	
Asp (D)	1	0.9	0	13	5	18	2.3333	0		9	11	20	3	
Cys (C)	8	7.3	1	4	2	7	2.266	0		3	2	5	0.73	
Gln (Q)	21	19.1	0	63	16	79	9.9	0		49	12	61	7.86	
Glu (E)	6	5.5	0	9	12	21	3.1	0		11	8	19	2.7	
Gly (G)	6	5.5	2	35	11	48	8.766	1		39	13	53	8.3	
His (H)	2	1.8	1	6	2	9	2.466	1		8	1	10	2.53	
Ile (I)	6	5.5	0	15	14	29	4.1667	2		10	12	24	6.16	
Leu (L)	6	5.5	6	18	18	42	13.9	5		18	18	41	12.53	
Lys (K)	5	4.5	1	5	5	11	2.866	0		8	7	15	2.16	
Met (M)	1	0.9	1	5	4	10	2.7	1		3	2	6	2.16	
Phe (F)	6	5.5	1	7	5	13	3.1	2		13	5	20	5.3	
Pro (P)	10	9.1	1	21	7	29	5.0333	0		17	8	25	3.4	
Ser (S)	4	3.6	0	16	9	25	3.3667	4		17	17	38	10.76	
Thr (T)	3	2.7	1	7	14	22	4.7	2		6	11	19	5.53	
Trp (W)	2	1.8	0	4	1	5	0.633	0		3	2	5	0.73	
Tyr (Y)	2	1.8	0	6	6	12	1.733	1		4	6	11	2.96	
Val (V)	5	4.5	5	19	15	39	12	1		19	14	34	6.13	
Pyl (O)	0	0	0	0	0	0	0	0		0	0	0	0	
Sec (U)	0	0	0	0	0	0	0	0		0	0	0	0	
Number of amino acids	110		23	296	190		99.9	23		283	190		99.96	
Molecular weight	12,690.53		2,408.04	32,938.47	21,192.11			2,541.05		3,0825.06	2,0960.66			
Theoretical pI	8.71		8	7.17	6.94			8.52		7.95	8.6			
Total number of negatively charged sesidues (Asp + Glu)	7		0	22	17			0		20	19			
Total number of Positively charged sesidues (Arg + Lys)	77		1	22	17			1		21	21			
Atomic composition														
C	557		113	1,415	929			119		1,337	917			
H	864		191	2,222	1,500			190		2,073	1,475			
N	164		27	446	268			28		415	267			
O	159		26	448	286			31		417	287			
S	9		2	9	6			1		6	4			
Formula	C_557_H_864_N_164_O_159_S_9_		C_113_H_191_N_27_O_26_S_2_	C_1415_H_2222_N_268_O_448_S_9_	C_929_H_1500_N_268_O_286_S_6_			C_119_H_190_N_28_O_31_S_1_		C_1337_H_2073_N_415_O_417_S_6_	C_917_H_1475_N_267_O_287_S_6_			
Total Number of atoms	1,753		359	4,540	2,989			369		4,248	2,950			
Aliphatic index	61.18		173.48	65.81	97			140		63.71	90.84			
GRAVY	−0.661		1.778	−0.903	−0.226			1.274		−0.753	−0.326			

GRAVY, Grand average of hydrophobicity.

### Protein Characterization

The purity of the napin and cruciferin proteins were confirmed by 1D-SDS-PAGE analysis. Napin migrated as a doublet in the range of 5–10 kDa, whereas cruciferin migrated as double triplets, the upper one with protein bands in the range of 25-35 kDa and the lower one with bands in the range of 15-20 kDa (**Figure 1**). The protein electrophoretic profile matches those from earlier reports (Höglund et al., 1992; Wanasundara, 2011; Stone et al., 2014; Akbari and Wu, 2015; Perera et al., 2016; Joehnke et al., 2019; Xu et al., 2019; Rahman et al., 2020) (**Supplementary Figure 2A**).

SDS-PAGE resolved proteins were electro-blotted onto nitrocellulose membrane for antibody detection by Western blotting. Napin large chain subunit was detected at approximately 9 kDa and the small subunit slightly below 4 kDa (Gruis et al., 2002; Perera et al., 2016; Joehnke et al., 2019). The intact mature napin protein was also detected. Cruciferin was detected as a single band around approximately 42 kDa due to the specificity of the antibody (Job et al., 2005; Lin et al., 2013; De Meyer et al., 2020) (**Supplementary Figure 2B**). Results indicated that the proteins were pure, and were not degraded or truncated.

### Antibacterial Activity Screening

AST had confluent bacterial growth on the agar plates and in growth control wells (1: 1 CAMHB: inoculum), indicating that all the bacteria grew optimally. The success of the disc diffusion assay was confirmed by the positive control antibiotics showing the expected zones of inhibition ≥14 mm (1.25 µg/23.75 µg of Trimethoprim/Sulfamethoxazole) and ≥26 mm (5µg Ciprofloxacin), as per the EUCAST guidelines. The water blanks showed no zones of inhibition, indicating that the water solvent was not contaminated with any antimicrobial substance. In the microdilution assays, a more quantitative assay than the disc diffusion AST, the positive controls showed the expected bacterial growth inhibition with ampicillin or gentamicin, as per the CLSI guidelines, indicating the assay success. *Enterobacter cloacae* was an exception, demonstrating resistance at the highest concentration of ampicillin (128 μg/ml). The CAMBH blank had no bacterial growth (absorbance <0.1), indicating no microbial contamination. The growth controls with CAMBH, inoculum and DMSO (1% or 2%), showed absorbance similar to the growth control CAMHB and inoculum only), indicating that the final DMSO concentration did not inhibit bacterial growth.

**Figure 4:**
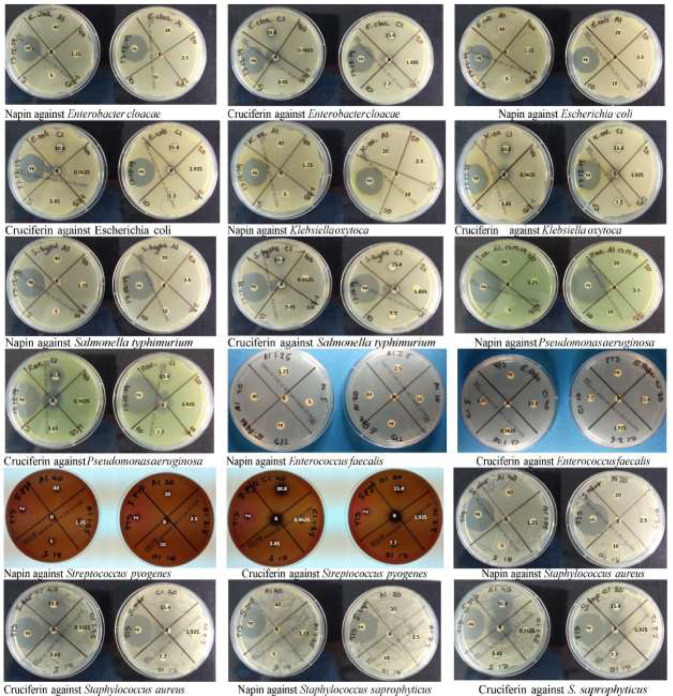
Plate images of disc diffusion antimicrobial susceptibility testing as per EUCAST guidelines for napin and cruciferin proteins against four Gram positive and five Gram negative bacteria, labeled electronically to reflect the actual concentrations tested. The agar plates used for the testing of the purified napin protein are labeled with the doses corresponding to 40 µg down to 1.25 µg, while plates used to test the purified cruciferin protein are labeled with the doses corresponding to 30.8 µg down to 0.9625 µg. The plates were labeled prior to the solubilization of the proteins (and subsequently, lower working concentrations were prepared for cruciferin 1, due to additional solvents added for solubilization). +v indicates that positive control antibiotics showing the expected zones of inhibition ≥14 mm (1.25 µg/23.75µg of Trimethoprim/Sulfamethoxazole) and ≥26 mm (5-µg Ciprofloxacin). B at the center indicates the blank negative control.

**Figure 5:**
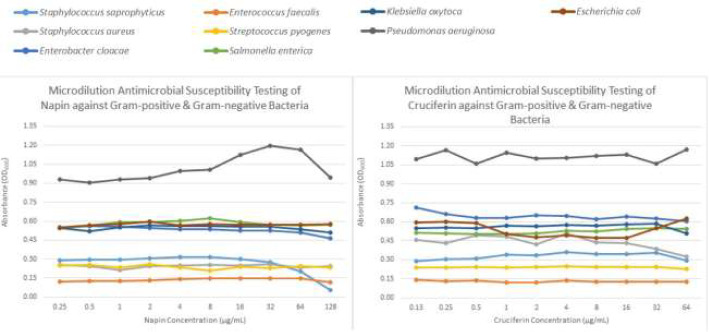
Graph showing the antimicrobial activity of napin and cruciferin against four Gram positive and five Gram negative bacteria, measured in microdilution antimicrobial susceptibility tests according to CLSI guidelines. The colors representing each bacterium are given at the top of the graph.

Napin did not show any antimicrobial activity in the disc diffusion AST, as no zones of inhibition were observed for any of the nine bacteria tested, even at the highest dose of 40 µg (concentration 40 µg/20 µl) (**Figure 4**). It did, however, show antimicrobial activity against *Staphylococcus saprophyticus* at 32, 64, and 128 μg/ml, that was dose dependent (**Figure 5**). Plating these test wells to Blood MH agar showed that the activity was bacteriostatic, not bactericidal, as *S. saprophyticus* growth was evident on the plates for every napin concentration inoculated. No antimicrobial activity for any napin concentration against any other bacteria tested was evident (**Figure 5**).

For cruciferin, only very small zones of inhibition against all nine bacteria tested were evident, however the activity was not greater than that of the solvent negative control (**Figure 4**). The solvent was tested in a growth control in the microdilution AST [CAMBH, inoculum and 8.7% acetic acid: acetonitrile: water (1:2:7)] and showed absorbance similar to the blank, confirming that this solvent, at this concentration, does inhibit bacterial growth. No antimicrobial activity for cruciferin was evident for all concentrations, against all bacteria tested (**Figure 5**).

**Figure 6:**
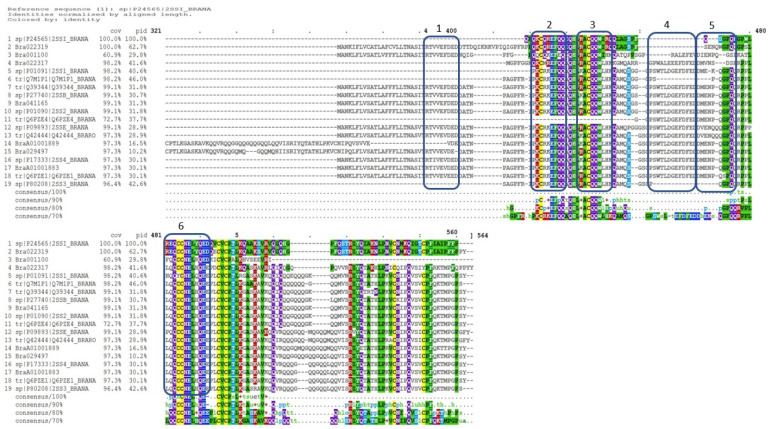
Multiple sequence alignment of *Brassica napus* napin proteins available in publicly open databases (**Supplementary Table 1**) and napin proteins identified in *Brassica rapa* R-o-18 aligned using the program Clustal Omega [Rahman et al., 2016; Rahman et al., 2017; Rahman et al., 2018a; Rahman et al., 2020; Rahman, 2020 (in preparation)], indicates significant identity of the two proteins. Cov, sequence coverage; pid, percent identity.

While the work carried out in this study focused on sequences and purified proteins from *B. napus* multiple sequence alignment of the napin sequences from *B. napus* against those of *B. rapa* indicated significant sequence identity between them (up to 100%) (**Figure 6**) with all motifs identified shared among the proteins (Rahman et al., 2020). This indicates that both the structural and characteristic antimicrobial properties of *B. napus* are highly likely to be retained in *B. rapa* napins. Further work to obtain purified napins from *B. rapa* and perform AST would confirm this.

## Discussion

Both napin and cruciferin storage proteins are synthesized during seed maturation in embryos (Höglund et al., 1992; Ellerström et al., 1996), and while providing a source of energy for the emerging seedling, these proteins may have additional roles, including defending the developing embryo against phytopathogenic bacteria and fungi. This potential antimicrobial activity is supported by the traditional use of mustard as an antimicrobial in food production. However, whether this activity is a function of the proteins or other compounds in the seeds (such as glucosinolates) remains to be elucidated. In order to provide experimental evidence to support the claims of *B. napus* napins and cruciferins having antimicrobial activity, *in silico* analysis of the protein sequences was carried out and *in vitro* antimicrobial activity of the purified proteins was tested.

Analysis of rapeseed napin sequences (**Supplementary Table 1**) in the PRINT database indicated conservation of specific signatures to sweet protein mabinlin-2 (P30233) and sweet protein mabinlin-4 (P80353) from *C. masaikai* (**Table 2**). These two proteins are related to the known antimicrobial peptide Flo (P24303) from *Moringa oleifera* (**Supplementary Figure 1A**) (Fisch et al., 2004; Suarez et al., 2005), a cationic polypeptide with flocculating properties that has been shown to destroy the cell membranes of bacteria (Garcia et al., 2019). In addition, Allergen Sin a 1, a napin homolog from *S. alba* was found to have functional similarity to *C. masaikai* in the PRINTS database search which is the first observation of possible antimicrobial functionality for this protein. Sin a 1 has been shown to be related to *B. rapa and B. napus* napins at the sequence level (Rahman et al., 2020). The database search also showed that rapeseed cruciferins (**Supplementary Table 1**) have high similarity with rice glutelins which have been reported as antimicrobial peptides (Bundó et al., 2014), as well as the antibacterial glycinin protein from *G. max* (Sitohy et al., 2012; Yang et al., 2016) and antibacterial Legumin A (P09802) from *Gossypium hirsutum* L. (**Supplementary Figure 1B**) (He et al., 2018). These results suggested the napin and cruciferin are most likely to have antimicrobial properties as well.

Previous reports have suggested that antimicrobial peptides act against a wide range of infectious bacterial and fungal strains, even against those resistant to multiple common antibiotics. Many of the antimicrobial peptides interact with the microbial membranes at hydrophobic and positively charged regions (Fisch et al., 2004). Antimicrobial peptides are also found to possess anionic function within their amphiphilic structures which enable interaction with membranes. These regions diverge from the traditional α-helical peptides to form a cyclic-cysteine-knot conformation found in some plant proteins (Harris et al., 2009; Seong and Hak, 2013). Anionic proteins usually interact with membranes through electrostatic interactions either by repulsion or formation of soluble or insoluble complexes based on charge state (Rommi et al., 2015). Napin is a very hydrophilic protein (Amine et al., 2019), whereas cruciferin has the ability to form soluble complexes with negatively charged carbohydrates on the microbial membrane surface (Rommi et al., 2015).

The amino acid sequence, conserved sequence motifs, and the number of cysteine residues and their spacing are often used as a basis to classify antimicrobial peptides (Nawrot et al., 2014). Napins show the characteristic conserved skeleton of cysteine-knot conformation of C-Xn-C-Xn-CC-Xn-CXC-Xn-C-Xn-C (Shewry et al., 2002) (**Supplementary Figure 1A**). Interestingly, all proteins demonstrating antimicrobial activity, namely, 2S albumin-like proteins, lipid transfer proteins, puroindolines, and thionins share a similar conserved skeleton of cysteines and possibly also a similar conformational folding pattern (Gautier et al., 1994).

Protein motifs are highly conserved during evolution, and it is postulated that the sequence of amino acids in the ligand binding sites to the receptors would remain conserved as well. The nature of binding, the binding pattern and binding energy between motif and receptor can provide evidence for functional interactions (Rajasekaran et al., 2008). To investigate these properties for napins and cruciferins from rapeseed, to bacterial target molecules, a molecular docking study was performed using the rapeseed 2S napin (1PNB) and 11S procruciferin (3KGL, as closest available 3D structure of 12S cruciferin) from *B. napus* as ligands. The docked models indicated that, like the earlier antimicrobial peptides, the proteins showed distinct amphipathic properties, cruciferin showed an amphipathic β-helical dominated structure while napin showed an amphipathic α helix dominated structure (Chang et al., 2015; Perera et al., 2016). The cysteine-based motif, conserved cysteine skeleton CXnCXnCCXnCXCXnCXnC held together by four disulfide bonds where C represents cysteine residues and Xn could be any number of other amino acids are found at positions 64, 76, 240, 241, 252, 254, 310, and 319 in the alignment (**Supplementary Figure 1A**, color-coded in yellow), can act like a hinge (Chang et al., 2015). The conserved cysteine residues at the edges of the cysteine-based motif are able to form disulfide bridges to the cysteine residues of other distant subunits; while the nearby glycine residue could provide the hinge region motif more flexibility (Chang et al., 2015).

The characteristic conserved cysteine-based motifs of 2S albumin-like napins are often used as fingerprints and exploited to characterise the prolamin superfamily (Shewry et al., 2002; Sharma et al., 2017). These motifs encode protein folds and provide flexibly to the structure. These motifs were identified as having similarity in the SPRINT database with previously reported antimicrobial peptides (**Supplementary Table 1** and **Supplementary Figures 1A, B**). In earlier studies, it was found that the bactericidal activity is associated to a specific sequence motif of amino acid loops, like cysteine-cysteine or proline-proline loops that are stabilised by formation of disulphide bridges. These have a tendency to form a helix-loop-helix conformation and this motif causes bacterial membrane damage (Suarez et al., 2005). Because napin type 2S albumins possess the same assembly of several copies of this conformational motif into a branched peptide, it could be assumed that they may also exert their antibacterial action in the same way the *M. oleifera* seed protein does.

Interestingly, cruciferins showed high (>86%) coverage and significant pairwise sequence identity (28-32%) with soybean glycinin (**Supplementary Figure 1B**) (Ramlan et al., 2002; Sitohy et al., 2012). It is also evident there are two highly conserved cysteine residues at positions 71 and 114 in the alignment with many other conserved amino acid motifs (**Supplementary Figure 1B**, color-coded in yellow).

Earlier reports (Chang et al., 2015; Farkas et al., 2017; Mohan et al., 2019) analyzing the critical regions of different antimicrobial proteins and examining antimicrobial peptide databases suggested that presence and absence of amino acid, their number and arrangement are critical for antimicrobial proteins. These influence the secondary structure and charge of the protein, conserved protein domains, amphipathicity, peptide aggregation, gapless alignments to highly similar protein sequences, receptor binding and ultimately antimicrobial functionality. In addition, these studies report that glycine is the most abundant residue in the critical regions of antimicrobial peptides (Chang et al., 2015). Interestingly, napins possess six glycine residues that enable the cysteine rich hinge motif higher flexibility, whereas cruciferins have much higher number of glycine residues (**Table 4**).

While *in silico* bioinformatics analysis of napin and cruciferin protein sequences suggested features and sequence motifs that have been attributed to anti-microbial proteins, and docking studies provided evidence for the ability of the proteins to bind to microbial proteins with high binding energy values, docking scores, and protein-receptor interactions (Barakat et al., 2016), the *in vitro* functional tests carried out in this study only demonstrated antimicrobial activity for napin against *S. saprophyticus* (**Figure 5**). This activity was bacteriostatic, not bactericidal, as the napin-induced growth inhibition microdilutions, when inoculated to Blood MH agar, grew *S. saprophyticus*, indicating bacterial viability when removed from the inhibiting effects of napin. The napin antimicrobial activity observed here provides evidence to warrant the further investigation of its antimicrobial activity using increased concentrations, with different extraction and purification approaches, and against different microorganisms. The bacteria *Klebsiella oxytoca* and *Enterobacter cloacae* used in this AST are intrinsically resistant to ampicillin. Napin exhibited strong antimicrobial activity against *S. saprophyticus* indicating a possible alternative to control bacteria.

In the case of cruciferin, while the zones of inhibition were not beyond that of the blank negative control (acetic acid/acetonitrile/water), indicating poor antimicrobial activity, there is the possibility that activity was masked by the solvent’s innate antimicrobial activity. The inclusion of this solvent in a growth control in the microdilution AST confirmed its antimicrobial activity, rendering it a poor solvent for AST. Therefore, cruciferin dissolved in 2% DMSO was applied in the microdilution AST (final concentration 1% DMSO). The 1% and 2% DMSO growth controls did not inhibit microbial growth; therefore, the lack of antimicrobial activity of cruciferin at the tested concentrations was confirmed. Higher concentrations need to be tested to determine the limit of detection for antimicrobial activity for both cruciferin and napin.

The *in vitro* activity we have shown for napin is somewhat contrary to what was recently reported for a napin protein from *B. juncea* (BjN), which showed strong inhibition of growth of *Xanthomonas oryzae* and *Staphylococcus aureus* at 60 µg, in disc diffusion AST with 0.1 mM Tris buffer solvents in Luria-Bertani medium (Munir et al., 2019). Antimicrobial activity of cruciferin and stronger antimicrobial activity of napin may be evident if higher concentrations of the proteins are used in the disc diffusion and microdilution AST, or the antimicrobial action of these proteins may be pH dependent, requiring different bacterial media with favorable diffusion and buffer solvents to maintain an optimal pH.

Several factors may play an important role behind these discrepancies and could relate to the initial protein extraction process, purification methods, stability of the protein, and even the method used for the disc diffusion functional assays. In this study, purified napin and cruciferin from *Brassica napus*, purchased from a commercial source, were used to evaluate the antimicrobial activity. These proteins were purified by simple ion exchange chromatography, but it is unknown how the initial extraction process was carried out, and what intermediary purification procedures were followed. This method of extraction may result in changes to protein functionality as low pH can cause the formation of protein aggregates (Wanasundara, 2011). Moreover, earlier studies showed cruciferin was unstable at low pH and high temperature, affecting its solubility, and causing unfolding of the protein (Perera et al., 2016).

Another factor that may play a role is the specific media used for the plate diffusion assay. The diffusion properties of napin and cruciferin through the Mueller Hinton agar media are unknown. The disc diffusion method works on the principle of the molecule being able to readily diffuse through agar to form a concentration gradient. The recent report on the antibacterial activity of napin used Luria-Bertani medium, as well as different methods for protein solubilization and application (Munir et al., 2019). The dose-dependent zones of inhibition observed for cruciferin in this study (**Figure 4**), that were only a few millimeters around the disc circumference, are attributed to the solvent used (acetic acid/acetonitrile/water), rather than the antibacterial activity of the protein, as the solvent concentration decreases with increasing dilutions, and the zones of inhibition decreased with each dilution. Furthermore, this solvent, in the absence of cruciferin, demonstrated strong antimicrobial activity against all bacteria tested in the microdilution AST.

One question that remains to be answered is how these SSPs exert their antimicrobial action. Evidence has shown that this could occur through different actions, including growth inhibition of bacteria, with the proteins inducing plasma membrane permeabilization leading to loss of integrity of the cell (Ribeiro et al., 2012). Additionally, antimicrobial proteins can induce production of nitric oxide in diverse pathogenic and non-pathogenic microorganisms (Ribeiro et al., 2012), or increase the production of reactive oxygen species (Garcia et al., 2019), leading to cell death. Some mammalian proteins are chemically converted to antimicrobial peptides inside the animal body. For example, truncated α-defensins ligate among themselves in the primate leukocytes and produce cyclic antimicrobial peptides which act against both bacteria and fungi even in low micromolar concentrations (Tang et al., 1999). It would be worth investigating the chemical modifications the studied peptides undergo and the biological activities of such chemically modified peptides in biological systems. Further work to elucidate this mode of action is needed. In this study, the proteins were tested against common laboratory microorganisms used for AST. Cruciferin and napin could be applied against a wider spectrum of disease-causing microorganisms for human, livestock and crops to check if they could be useful in the management of pathogen-borne diseases.

## Conclusion

This study evaluated the evidence for the role of rapeseed SSPs, napin, and cruciferin as antimcrobial agents. Similarity with other plant antimicrobial peptides through conservation of sequence motifs and specific amino acids, as well as 3D structural analysis, was presented. The results support further functional studies into the potential application of napin and cruciferin as potent candidates for antimicrobial agents, as well as functional food ingredient and in complementary medicine to alleviate diseases, as preservative for wide range of foods as well as crop protection from pathogens. Among a range of bacterial species tested, only napin demonstrated biological activity against *Staphylococcus saprophyticus*. The evidence presented here supports further investigation of the antimicrobial activity of napin and cruciferin for their potential application in the health, food, and agricultural industries.

## Data Availability Statement

The raw data supporting the conclusions of this article will be made available by the authors, without undue reservation, to any qualified researcher.

## Author Contributions

MR, MH, JB, JC, LL, and BB contributed substantially with data analysis and discussions of the results. MR conceived the study, designed the experiments, and wrote the article. JB, JC, and MR carried out the agar plate disc diffusion antimicrobial susceptibility tests and analysis. JB and JC carried out the microdilution antimicrobial susceptibility tests and analysis. MH extracted the molecular docking results. BB and LL supervised the study, edited, and reviewed the manuscript before submission.

## Funding

MR received support from an International Postgraduate Research Scholarship (IPRS) and Australian Postgraduate Award scholarship (APA), funded by the Australian Government to pursue his PhD from February 2016 to July 2019 and this article is a part of his PhD study. This study did not obtain any other external grant from funding bodies.

## Conflict of Interest

The authors declare that the research was conducted in the absence of any commercial or financial relationships that could be construed as a potential conflict of interest.

The handling editor declared a shared affiliation, though no other collaboration, with one of the authors, MH, at time of review.

## Abbreviations

1D-SDS-PAGE, One Dimensional Sodium Dodecyl Sulfate -Polyacrylamide Gel Electrophoresis; 3D**-**structure, Three Dimensional structure; ACE, Atomic Contact Energy; AMP and AMPed, Antimicrobial Peptide Databases; AST, Antimicrobial Susceptibility Test; ATCC, American Type Culture Collection; CAMHB, Cation-Adjusted Mueller-Hinton Broth; CLSI, Clinical and Laboratory Standards Institute; DMSO, Dimethyl Sulfoxide; EUCAST, The European Committee on Antimicrobial Susceptibility Testing; GRAVY, Grand average of hydrophobicity; MH, Mueller Hinton; SSP, Seed storage proteins.

## References

[B1] Aamir M. Singh V. K. Dubey M. K. Meena M. Kashyap S. P. Katari S. K. (2018). In silico Prediction, Characterization, Molecular Docking, and Dynamic Studies on Fungal SDRs as Novel Targets for Searching Potential Fungicides Against Fusarium Wilt in Tomato. Front. Pharmacol. 9, 1–28. 10.3389/fphar.2018.01038 30405403 PMC6204350

[B2] Aboelsoud N. H. (2010). Herbal medicine in ancient Egypt. J. Med. Plants Res. 4, 082–086. 10.5897/JMPR09.013

[B3] Akbari A. Wu J. (2015). An integrated method of isolating napin and cruciferin from defatted canola meal. LWT-Food Sci. Technol. 64, 308–315. 10.1016/j.lwt.2015.05.046

[B4] Altschul S. F. Madden T. L. Schäffer A. A. Zhang J. Zhang Z. Miller W. (1997). Gapped BLAST and PSI-BLAST: a new generation of protein database search programs. Nucleic Acids Res. 25, 3389–3402. 10.1093/nar/25.17.3389 9254694 PMC146917

[B5] Amine C. Boire A. Kermarrec A. Renard D. (2019). Associative properties of rapeseed napin and pectin: Competition between liquid-liquid and liquid-solid phase separation. Food Hydrocolloids 92, 94–103. 10.1016/j.foodhyd.2019.01.026

[B6] Andrusier N. Nussinov R. Wolfson H. J. (2007). FireDock: Fast interaction refinement in molecular docking. Proteins: Struct. Funct. Bioinf. 69, 139–159. 10.1002/prot.21495 17598144

[B7] Attwood T. K. Bradley P. Flower D. R. Gaulton A. Maudling N. Mitchell A. L. (2003). PRINTS and its automatic supplement, prePRINTS acid pharmacophores. Nucleic Acids Res. 31, 400–402. 10.1093/nar/gkg030 PMC165477 12520033

[B8] Bai X. Chen Y. Liu Z. Zhang L. Zhang T. Feng B. (2019). Synthesis, Antimicrobial Activities, and Molecular Docking Studies of Dihydrotriazine Derivatives Bearing a Quinoline Moiety. Chem. Biodivers. 16, e1900056. 10.1002/cbdv.201900056 30957398

[B9] Barakat A. Al-Qahtani B. M. Al-Majid A. M. Shaik M. A. M. R. Al-Agamy M. H. Wadood A. (2016). Synthesis, characterization, antimicrobial activity and molecular docking studies of combined pyrazol-barbituric. Trop. J. Pharm. Res. 15, 2197–2207. 10.4314/tjpr.v15i10.19

[B10] Barkla B. J. Vera-Estrella R. Pantoja O. Kirch H.-H. Bohnert H. J. (1999). Aquaporin localization–how valid are the TIP and PIP labels? Trends Plant Sci. 4, 86–88. 10.1016/s1360-1385(99)01388-6 10322537

[B11] Barkla B. J. Vera-Estrella R. Raymond C. (2016). Single-cell-type quantitative proteomic and ionomic analysis of epidermal bladder cells from the halophyte model plant *Mesembryanthemum crystallinum* to identify salt-responsive proteins. BMC Plant Biol. 16. 10.1186/s12870-016-0797-1 PMC4862212 27160145

[B12] Batool M. Tajammal A. Farhat F. Verpoort F. Khattak Z. Shahid M. (2018). Molecular Docking, Computational, and Antithrombotic Studies of Novel 1, 3, 4-Oxadiazole Derivatives. Int. J. Mol. Sci. 19, 3606. 10.3390/ijms19113606 PMC6274789 30445728

[B13] Borges A. Abreu A. C. Ferreira C. Saavedra M. J. Simões L. C. Simões M. (2015). Antibacterial activity and mode of action of selected glucosinolate hydrolysis products against bacterial pathogens. J. Food Sci. Technol. 52, 4737–4748. 10.1007/s13197-014-1533-1 26243895 PMC4519465

[B14] Breiteneder H. Radauer C. (2004). A classification of plant food allergens. J. Allergy Clin. Immunol. 113, 821–830. 10.1016/j.jaci.2004.01.779 15131562

[B15] Bundó M. Montesinos L. Izquierdo E. Campo S. Mieulet D. Guiderdoni E. (2014). Production of cecropin A antimicrobial peptide in rice seed endosperm. BMC Plant Biol. 14, 102. 10.1186/1471-2229-14-102 24755305 PMC4032361

[B16] Chang K. Y. Lin T.-P. Shih L.-Y. Wang C.-K. (2015). Analysis and prediction of the critical regions of antimicrobial peptides based on conditional random fields. PloS One 10, e0119490–e0119490. 10.1371/journal.pone.0119490 25803302 PMC4372350

[B17] De Meyer T. Arcalis E. Melnik S. Maleux K. Nolf J. Altmann F. (2020). Seed-produced anti-globulin VHH-Fc antibodies retrieve globulin precursors in the insoluble fraction and modulate the Arabidopsis thaliana seed subcellular morphology. Plant Mol. Biol. 103, 597–608. 32346812 10.1007/s11103-020-01007-w

[B18] Deleu M. Vaca-Medina G. Fabre J.-F. Roïz J. Valentin R. Mouloungui Z. (2010). Interfacial properties of oleosins and phospholipids from rapeseed for the stability of oil bodies in aqueous medium. Colloids Surfaces B: Biointerfaces 80, 125–132. 10.1016/j.colsurfb.2010.05.036 20580539

[B19] Deng Y. Tang D. Wang Q.-R. Huang S. Fu L.-Z. Li C.-H. (2019). Semi-synthesis, antibacterial activity, and molecular docking study of novel pleuromutilin derivatives bearing cinnamic acids moieties. Archiv. der Pharmazie 352, 1800266. 10.1002/ardp.201800266 30536467

[B20] Duhovny D. Nussinov R. Wolfson H. J. (2002). “Efficient unbound docking of rigid molecules,” in International workshop on algorithms in bioinformatics (Berlin, Heidelberg: Springer), 185–200.

[B21] Ebrahimi S. Nikkhah A. Sadeghi A. Raisali G. (2009). Chemical composition, secondary compounds, ruminal degradation and in vitro crude protein digestibility of gamma irradiated canola seed. Anim. feed Sci. Technol. 151, 184–193. 10.1016/j.anifeedsci.2009.01.014

[B22] Ellerström M. Stålberg K. Ezcurra I. Rask L. (1996). Functional dissection of a napin gene promoter: identification of promoter elements required for embryo and endosperm-specific transcription. Plant Mol. Biol. 32, 1019–1027. 10.1007/BF00041385 9002600

[B23] Farkas A. Maróti G. Kereszt A. Kondorosi É. (2017). Comparative Analysis of the Bacterial Membrane Disruption Effect of Two Natural Plant Antimicrobial Peptides. Front. Microbiol. 8, 1–12. 10.3389/fmicb.2017.00051 28167938 PMC5253368

[B24] Fisch F. Suarez M. Mermoud N. (2004). Flo antibacterial peptide from the tropical tree Moringa oleifera: A template for novel antibacterial agents. Travail diploma Universite Lausanne Lausanne fevrier 12 (1-2), 1–4.

[B25] Garcia T. B. Soares A. A. Costa J. H. Costa H. P. S. Neto J. X. S. Rocha-Bezerra L. C. B. (2019). Gene expression and spatiotemporal localization of antifungal chitin-binding proteins during Moringa oleifera seed development and germination. Planta 249, 1503–1519. 10.1007/s00425-019-03103-8 30706136

[B26] Gautier M. F. Aleman M. E. Guirao A. Marion D. Joudrier P. (1994). Triticum aestivum puroindolines, two basic cystine-rich seed proteins: cDNA sequence analysis and developmental gene expression. Plant Mol. Biol. 25, 43–57. 10.1007/BF00024197 7516201

[B27] Gruis D. F. Selinger D. A. Curran J. M. Jung R. (2002). Redundant proteolytic mechanisms process seed storage proteins in the absence of seed-type members of the vacuolar processing enzyme family of cysteine proteases. Plant Cell 14, 2863–2882. 10.1105/tpc.005009 12417707 PMC152733

[B28] Gullapelli K. Brahmeshwari G. Ravichander M. Kusuma U. (2017). Synthesis, antibacterial and molecular docking studies of new benzimidazole derivatives. Egyptian J. Basic Appl. Sci. 4, 303–309. 10.1016/j.ejbas.2017.09.002

[B29] Gupta M. Shaw B. (2009). Uses of medicinal plants in Panchakarma Ayurvedic therapy. Indian J. Trad. Knowledge 8, 372–378.

[B30] Gupta M. (2010). Pharmacological properties and traditional therapeutic uses of important Indian spices: A review. Int. J. Food Properties 13, 1092–1116. 10.1080/10942910902963271

[B31] Harris F. Dennison S. R. Phoenix D. A. (2009). Anionic antimicrobial peptides from eukaryotic organisms. Curr. Protein Pept. Sci. 10, 585–606. 10.2174/138920309789630589 19751192

[B32] He Z. Zhang D. Cao H. (2018). Protein profiling of water and alkali soluble cottonseed protein isolates. Sci. Rep. 8, 9306–9306. 10.1038/s41598-018-27671-z 29915326 PMC6006339

[B33] Höglund A.-S. Rödin J. Larsson E. Rask L. (1992). Distribution of napin and cruciferin in developing rape seed embryos. Plant Physiol. 98, 509–515. 10.1104/pp.98.2.509 16668669 PMC1080218

[B34] Jia J. Wu Q. Yan H. Gui Z. (2015). Purification and molecular docking study of a novel angiotensin-I converting enzyme (ACE) inhibitory peptide from alcalase hydrolysate of ultrasonic-pretreated silkworm pupa (*Bombyx mori*) protein. Process Biochem. 50, 876–883. 10.1016/j.procbio.2014.12.030 25111373

[B35] Job C. Rajjou L. Lovigny Y. Belghazi M. Job D. (2005). Patterns of protein oxidation in Arabidopsis seeds and during germination. Plant Physiol. 138, 790–802. 10.1104/pp.105.062778 15908592 PMC1150397

[B36] Joehnke M. S. Sørensen S. Bjergegaard C. Markedal K. E. Sørensen J. C. (2018). Effect of dietary fibre fractions on in vitro digestibility of rapeseed napin proteins. Polish J. Food Nutr. Sci. 68, 335–345. 10.2478/pjfns-2018-0005

[B37] Joehnke M. S. Lametsch R. Sørensen J. C. (2019). Improved in vitro digestibility of rapeseed napin proteins in mixtures with bovine beta-lactoglobulin. Food Res. Int. 123, 346–354. 10.1016/j.foodres.2019.05.004 31284985

[B38] Jyothi T. Sinha S. Singh S. A. Surolia A. Rao A. A. (2007). Napin from *Brassica juncea*: Thermodynamic and structural analysis of stability. Biochim. Biophys. Acta (BBA)-Proteins Proteomics 1774, 907–919. 10.1016/j.bbapap.2007.04.008 17544981

[B39] Kabir S. M. R. Rahman M. Khatun A. Saha S. Roy A. Rashid A. H. M. A. (2016). Total flavonoids content and reducing power assay of twelve common Bangladeshi leafy vegetables. PharmacologyOnline 2016, 6–14.

[B40] Kasprzak M. Houdijk J. Liddell S. Davis K. Olukosi O. Kightley S. (2016). Rapeseed napin and cruciferin are readily digested by poultry. J. Anim. Physiol. Anim. Nutr. 101, 558–666. 10.1111/jpn.1257 27562881

[B41] Khan S. A. Shahid S. Jameel M. Ahmad A. (2016). In vitro antibacterial, antifungal and GC-MS analysis of seeds of Mustard Brown. Int. J. Pharm. Chem. 6, 107–115. 10.7439/ijpc.v6i4.3185

[B42] Lin Y. Pajak A. Marsolais F. Mccourt P. Riggs C. D. (2013). Characterization of a cruciferin deficient mutant of Arabidopsis and its utility for overexpression of foreign proteins in plants. PloS One 8, e64980. 10.1371/journal.pone.0064980 23724110 PMC3664629

[B43] Liu L. Liu T. Li G. Wang Q. Ng T. (2003). Isolation and determination of p-hydroxybenzoylcholine in traditional Chinese medicine Semen sinapis Albae. Anal. Bioanal. Chem. 376, 854–858. 10.1007/s00216-003-1964-4 12811446

[B44] Madeira F. Park Y. M. Lee J. Buso N. Gur T. Madhusoodanan N. (2019). The EMBL-EBI search and sequence analysis tools APIs in 2019. Nucleic Acids Res. 47, W636–W641. 10.1093/nar/gkz268 30976793 PMC6602479

[B45] Maria-Neto S. Honorato R. V. Costa F. T. Almeida R. G. Amaro D. S. Oliveira J. T. A. (2011). Bactericidal activity identified in 2S Albumin from sesame seeds and in silico studies of structure-function relations. Protein J. 30, 340–350. 10.1007/s10930-011-9337-x 21691771

[B46] Mazzio E. Badisa R. Eyunni S. Ablordeppey S. George B. Soliman K. F. A. (2018). Bioactivity-guided isolation of neuritogenic factor from the seeds of the Gac plant (*Momordica cochinchinensis*). Evidence-Based Complement. Altern. Med. 2018, 8953958–8953958. 10.1155/2018/8953958 PMC6000838 29955238

[B47] Mcinnis S. M. (1998). The isolation and molecular characterization of a 2S albumin gene from Picea glauca (Vancouver, Canada: University of British Columbia).

[B48] Mir S. A. Shah M. A. Mir M. M. (2017). Microgreens: Production, shelf life, and bioactive components. Crit. Rev. Food Sci. Nutr. 57, 2730–2736. 10.1080/10408398.2016.1144557 26857557

[B49] Mohan N. M. Zorgani A. Jalowicki G. Kerr A. Khaldi N. Martins M. (2019). Unlocking NuriPep 1653 From Common Pea Protein: A Potent Antimicrobial Peptide to Tackle a Pan-Drug Resistant Acinetobacter baumannii. Front. Microbiol. 10, 1–16. 10.3389/fmicb.2019.02086 31620099 PMC6759681

[B50] Munir A. Iqbal S. Khaliq B. Saeed Q. Hussain S. Shah K. H. (2019). In Silico Studies and Functional Characterization of a Napin Protein from Seeds of *Brassica juncea* . Int. J. Agri. Biol. 22, 1655–1662. 10.17957/IJAB/15.1247

[B51] Nawrot R. Barylski J. Nowicki G. Broniarczyk J. Buchwald W. Goździcka-Józefiak A. (2014). Plant antimicrobial peptides. Folia Microbiol. 59, 181–196. 10.1007/s12223-013-0280-4 24092498 PMC3971460

[B52] Ngai P. Ng T. (2004). A napin-like polypeptide from dwarf Chinese white cabbage seeds with translation-inhibitory, trypsin-inhibitory, and antibacterial activities. Peptides 25, 171–176. 10.1016/j.peptides.2003.12.012 15062997

[B53] Nietzel T. Dudkina N. V. Haase C. Denolf P. Semchonok D. A. Boekema E. J. (2013). The native structure and composition of the cruciferin complex in *Brassica napus* . J. Biol. Chem. 288, 2238–2245. 10.1074/jbc.M112.356089 23192340 PMC3554896

[B54] Oferkin I. V. Katkova E. V. Sulimov A. V. Kutov D. C. Sobolev S. I. Voevodin V. V. (2015). Evaluation of Docking Target Functions by the Comprehensive Investigation of Protein-Ligand Energy Minima. Adv. Bioinf. 2015, 12. 10.1155/2015/126858 PMC4674582 26693223

[B55] Pacheco-Cano R. Salcedo-Hernández R. López-Meza J. Bideshi D. Barboza-Corona J. (2018). Antimicrobial activity of broccoli (Brassica oleracea var. italica) cultivar Avenger against pathogenic bacteria, phytopathogenic filamentous fungi and yeast. J. Appl. Microbiol. 124, 126–135. 10.1111/jam.13629 29112318

[B56] Perera S. P. Mcintosh T. C. Wanasundara J. P. (2016). Structural properties of cruciferin and napin of *Brassica napus* (Canola) show distinct responses to changes in pH and temperature. Plants 5, 36. 10.3390/plants5030036 PMC5039744 27618118

[B57] Pirtskhalava M. Gabrielian A. Cruz P. Griggs H. L. Squires R. B. Hurt D. E. (2015). DBAASP v. 2: an enhanced database of structure and antimicrobial/cytotoxic activity of natural and synthetic peptides. Nucleic Acids Res. 44, D1104–D1112. 10.1093/nar/gkv1174 26578581 PMC4702840

[B58] Pisano M. B. Kumar A. Medda R. Gatto G. Pal R. Fais A. (2019). Antibacterial Activity and Molecular Docking Studies of a Selected Series of Hydroxy-3-arylcoumarins. *Molecules (Basel* . Switzerland) 24, 2815. 10.3390/molecules24152815 PMC6696357 31375003

[B59] Rahman M. Liu L. King G. J. Barkla B. J. (2016). “Characterizing Brassica seed storage protein mutants to enhance the nutritional value of oilseed,” in Brassica 2016 (Melbourne, Australia: Australian Research Assembly on Brassicas (ARAB)).

[B60] Rahman M. Baten A. King G. J. Liu L. Barkla B. J. (2017). “Identification of 2S albumin type napin genes in the Brassica rapa genome,” in 67th Australasian Grain Science Conference (Christchurch, New Zealand: Australasian Grain Science Association).

[B61] Rahman M. Baten A. King G. J. Liu L. Pantoja O. Barkla B. J. (2018a). “Computational and biological characterization of 2S albumin proteins from Brassica rapa,” in AusCanola 2018, the 20th Australian Research Assembly on Brassicas (Scarborough, Perth, Western Australia: Australian Oilseeds Federation Inc. and Grain Industry Association of Western Australia Inc).

[B62] Rahman M. Khatun A. Liu L. Barkla B. J. (2018b). Brassicaceae mustards: Traditional and agronomic uses in Australia and New Zealand. Molecules 23 18 pages. 10.3390/molecules23010231 PMC6017612 29361740

[B63] Rahman M. Baten A. Mauleon R. King G. J. Liu L. Barkla B. J. (2020). Identification, characterization and epitope mapping of proteins encoded by putative allergenic napin genes from *Brassica rapa* . Clin. Exp. Allergy 50, 848–868. 10.1111/cea.13612 32306538

[B64] Rahman M. (2020). Identification, Molecular and Proteomic Characterisation of Brassica rapa Seed Storage Proteins with Allergenic and Antimicrobial Potential (Lismore, Australia: Doctor of Philosophy, Southern Cross University).

[B65] Rajasekaran S. Balla S. Gradie P. Gryk M. R. Kadaveru K. Kundeti V. (2008). Minimotif miner 2nd release: a database and web system for motif search. Nucleic Acids Res. 37, D185–D190. 10.1093/nar/gkn865 18978024 PMC2686579

[B66] Ramlan M. Maruyama N. Adachi M. Hontani N. Saka S. Kato N. (2002). Comparison of protein chemical and physicochemical properties of rapeseed cruciferin with those of soybean glycinin. J. Agric. Food Chem. 50, 7380–7385. 10.1021/jf0202537 12452662

[B67] Rehder A. Sulewska A. M. Markedal K. E. Sørensen S. Sørensen J. C. (2017). Solubility of a cruciferin-rich protein product purified from rapeseed pressed cake (Brassica napus L.) by an aqueous processing method. Int. J. Food Sci. Technol. 52, 1653–1659. 10.1111/ijfs.13446

[B68] Rehman T. U. Khan A.-U. Abbas A. Hussain J. Khan F. U. Stieglitz K. (2018). Investigation of nepetolide as a novel lead compound: Antioxidant, antimicrobial, cytotoxic, anticancer, anti-inflammatory, analgesic activities and molecular docking evaluation. Saudi Pharm. J. 26, 422–429. 10.1016/j.jsps.2017.12.019 29556134 PMC5856943

[B69] Ren C. Bewley J. D. (1999). Developmental and germinative events can occur concurrently in precociously germinating Chinese cabbage (*Brassica rapa* ssp. Pekinensis) seeds. J. Exp. Bot. 50, 1751–1761. 10.1093/jxb/50.341.1751

[B70] Riaz M. B. Khan A.-U. Qazi N. G. (2019). Pharmacological and computational evaluation of fig for therapeutic potential in hyperactive gastrointestinal disorders. BMC Complement. Altern. Med. 19, 348. 10.1186/s12906-019-2759-2 31796063 PMC6889615

[B71] Ribeiro S. F. F. Taveira G. B. Carvalho A. O. Dias G. B. Da Cunha M. Santa-Catarina C. (2012). Antifungal and Other Biological Activities of Two 2S Albumin-Homologous Proteins Against Pathogenic Fungi. Protein J. 31, 59–67. 10.1007/s10930-011-9375-4 22120089

[B72] Robin A. H. K. Hossain M. R. Park J.-I. Kim H. R. Nou I.-S. (2017). Glucosinolate profiles in cabbage genotypes Influence the preferential feeding of diamondback moth (*Plutella xylostella*). Front. Plant Sci. 8, 1244. 10.3389/fpls.2017.01244 28769953 PMC5513964

[B73] Rommi K. Ercili-Cura D. Hakala T. K. Nordlund E. Poutanen K. Lantto R. (2015). Impact of Total Solid Content and Extraction pH on Enzyme-Aided Recovery of Protein from Defatted Rapeseed (Brassica rapa L.) Press Cake and Physicochemical Properties of the Protein Fractions. J. Agric. Food Chem. 63, 2997–3003. 10.1021/acs.jafc.5b01077 25739320

[B74] Schäffer A. A. Aravind L. Madden T. L. Shavirin S. Spouge J. L. Wolf Y. I. (2001). Improving the accuracy of PSI-BLAST protein database searches with composition-based statistics and other refinements. Nucleic Acids Res. 29, 2994–3005. 10.1093/nar/29.14.2994 11452024 PMC55814

[B75] Schneidman-Duhovny D. Inbar Y. Nussinov R. Wolfson H. J. (2005). PatchDock and SymmDock: servers for rigid and symmetric docking. Nucleic Acids Res. 33, W363–W367. 10.1093/nar/gki481 15980490 PMC1160241

[B76] Schomburg K. (2014). Protein-Ligand Inverse Screening and its Application in Biotechnology and Pharmacology. (Germany: Universität Hamburg).

[B77] Seong M.-D. Hak Y.-I. (2013). Antimicrobial peptides properties, functions and role in immune response (New York: Nova Science Publishers, Inc).

[B78] Shang R. Wang S. Xu X. Yi Y. Guo W. Yuliu (2013). Chemical synthesis and biological activities of novel pleuromutilin derivatives with substituted amino moiety. PloS One 8, e82595. 10.1371/journal.pone.0082595 24376551 PMC3871055

[B79] Sharma A. Kumar P. Kesari P. Katiki M. Mishra M. Singh P. K. (2017). Purification and Characterization of 2S Albumin from Seeds of Wrightia tinctoria Exhibiting Antibacterial and DNase Activity. Protein Pept. Lett. 24, 368–378. 10.2174/0929866524666170126144936 28128054

[B80] Shewry P. Beaudoin F. Jenkins J. Griffiths-Jones S. Mills E. (2002). “Plant protein families and their relationships to food allergy” (Portland Press Limited). 10.1042/bst0300906 12440943

[B81] Shimada T. Fuji K. Tamura K. Kondo M. Nishimura M. Hara-Nishimura I. (2003a). Vacuolar sorting receptor for seed storage proteins in *Arabidopsis thaliana* . Proc. Natl. Acad. Sci. U States America 100, 16095–16100. 10.1073/pnas.2530568100 PMC307698 14657332

[B82] Shimada T. Yamada K. Kataoka M. Nakaune S. Koumoto Y. Kuroyanagi M. (2003b). Vacuolar processing enzymes are essential for proper processing of seed storage proteins in *Arabidopsis thaliana* . J. Biol. Chem. 278, 32292–32299. 10.1074/jbc.M305740200 12799370

[B83] Sitohy M. Z. Mahgoub S. A. Osman A. O. (2012). In vitro and in situ antimicrobial action and mechanism of glycinin and its basic subunit. Int. J. Food Microbiol. 154, 19–29. 10.1016/j.ijfoodmicro.2011.12.004 22236762

[B84] Stone A. K. Teymurova A. Dang Q. Abeysekara S. Karalash A. Nickerson M. T. (2014). Formation and functional attributes of electrostatic complexes involving napin protein isolate and anionic polysaccharides. Eur. Food Res. Technol. 238, 773–780. 10.1007/s00217-014-2159-2

[B85] Suarez M. Haenni M. Canarelli S. Fisch F. Chodanowski P. Servis C. (2005). Structure-function characterization and optimization of a plant-derived antibacterial peptide. Antimicrob. Agents Chemother. 49, 3847–3857. 10.1128/AAC.49.9.3847-3857.2005 16127062 PMC1195432

[B86] Tang Y.-Q. Yuan J. Ösapay G. Ösapay K. Tran D. Miller C. J. (1999). A Cyclic Antimicrobial Peptide Produced in Primate Leukocytes by the Ligation of Two Truncated α-Defensins. Science 286, 498–502. 10.1126/science.286.5439.498 10521339

[B87] Tenore G. C. Troisi J. Di Fiore R. Basile A. Novellino E. (2012). Chemical composition, antioxidant and antimicrobial properties of Rapa Catozza Napoletana (Brassica rapa L. var. rapa DC.) seed meal, a promising protein source of Campania region (southern Italy) horticultural germplasm. J. Sci. Food Agri. 92 (8), 1716–1724. 10.1002/jsfa.5537 22173690

[B88] Terras F. Schoofs H. De Bolle M. Van Leuven F. Rees S. B. Vanderleyden J. (1992). Analysis of two novel classes of plant antifungal proteins from radish (Raphanus sativus L.) seeds. J. Biol. Chem. 267, 15301–15309. 1639777

[B89] Terras F. R. Goderis I. J. Van Leuven F. Vanderleyden J. Cammue B. P. Broekaert W. F. (1992). In vitro antifungal activity of a radish (*Raphanus sativus* L.) seed protein homologous to nonspecific lipid transfer proteins. Plant Physiol. 100, 1055–1058. 10.1104/pp.100.2.1055 16653017 PMC1075666

[B90] Terras F. R. Torrekens S. Van Leuven F. Osborn R. W. Vanderleyden J. Cammue B. P. (1993). A new family of basic cysteine-rich plant antifungal proteins from Brassicaceae species. FEBS Lett. 316, 233–240. 10.1016/0014-5793(93)81299-F 8422949

[B91] Terras F. Torrekens S. Van Leuven F. Broekaert W. (1996). A six-cysteine type thionin from the radisch storage organ displays weak in vitro antifungal activity against *Fusarium culmorum* . Plant Physiol. Biochem. 34, 599–603.

[B92] Torrijos R. Nazareth T. M. Pérez J. Mañes J. Meca G. (2019). Development of a bioactive sauce based on oriental mustard flour with antifungal properties for PITA bread shelf life improvement. Molecules 24, 1019. 10.3390/molecules24061019 PMC6471135 30875724

[B93] Turner A. Radburn-Smith K. Mushtaq A. Tan L. (2011). Storage and Handling Guidelines for Custom Peptides. Curr. Protoc. Protein Sci. 64, 18.12.11–18.12.17. 10.1002/0471140864.ps1812s64 21488043

[B94] Von Der Haar D. Müller K. Bader-Mittermaier S. Eisner P. (2014). Rapeseed proteins–Production methods and possible application ranges. OCL 21, D104. 10.1051/ocl/2013038

[B95] Wanasundara J. P. D. (2011). Proteins of Brassicaceae oilseeds and their potential as a plant protein source. Crit. Rev. Food Sci. Nutr. 51, 635–677. 10.1080/10408391003749942 21793726

[B96] Wang G. Li X. Zasloff M. (2010). “A database view of naturally occurring antimicrobial peptides: nomenclature, classification and amino acid sequence analysis,” in Antimicrobial Peptides: Discovery, Design and Novel Therapeutic Strategies. CABI), (Oxfordshire, UK: CAB International) 1–21.

[B97] Wang G. Li X. Wang Z. (2015). APD3: the antimicrobial peptide database as a tool for research and education. Nucleic Acids Res. 44, D1087–D1093. 10.1093/nar/gkv1278 26602694 PMC4702905

[B98] Withana Gamage T. (2013). Structure and properties of cruciferin: investigation of homohexameric cruciferin expressed in Arabidopsis. (Saskatoon, Canada: University of Saskatchewan). 10.1093/nar/gkv1278

[B99] Withana-Gamage T. S. Hegedus D. D. Qiu X. Wanasundara J. P. D. (2011). In Silico Homology Modeling To Predict Functional Properties of Cruciferin. J. Agric. Food Chem. 59, 12925–12938. 10.1021/jf201979a 22077583

[B100] Wu J. Muir A. (2008). Comparative structural, emulsifying, and biological properties of 2 major canola proteins, cruciferin and napin. J. Food Sci. 73, C210–C216. 10.1111/j.1750-3841.2008.00675.x 18387101

[B101] Xu F. Yao Y. Xu X. Wang M. Pan M. Ji S. (2019). Identification and Quantification of DPP-IV-Inhibitory Peptides from Hydrolyzed-Rapeseed-Protein-Derived Napin with Analysis of the Interactions between Key Residues and Protein Domains. J. Agric. Food Chem. 67, 3679–3690. 10.1021/acs.jafc.9b01069 30854852

[B102] Yang X. Xiao Y. Wang X. Pei Y. (2007). Expression of a novel small antimicrobial protein from the seeds of motherwort (*Leonurus japonicus*) confers disease resistance in tobacco. Appl. Environ. Microbiol. 73, 939–946. 10.1128/AEM.02016-06 17158620 PMC1800757

[B103] Yang J. Sun G.-J. Li Y.-Q. Cui K.-Y. Mo H. Z. (2016). Antibacterial characteristics of glycinin basic polypeptide against Staphylococcus aureus. Food Sci. Biotechnol. 25, 1477–1483. 10.1007/s10068-016-0229-x 30263433 PMC6049286

