# Supplementary material for: In Silico, Molecular Docking and In Vitro Antimicrobial Activity of the Major Rapeseed Seed Storage Proteins
Source: Front Pharmacol. 2020 Sep 8;11:1340. doi: 10.3389/fphar.2020.01340 (PMC7508056; doi:10.3389/fphar.2020.01340)
Supplement: Supplementary file 1 [file DataSheet_1.docx]

**Supplementary Table 1:** 2S albumins and 12S globulins with known and unknown antimicrobial activity

| Sl | Protein name | Species | Peptide length | Sequence (Uniprot entry is followed by >sp\|) | | F/C |
| --- | --- | --- | --- | --- | --- | --- |
| 2S albumins with known antimicrobial activity | | | | |  |  |
| 1 | 2S albumin | *Glycine max* (Soybean) | 158 | >sp\|P19594\|2SS_SOYBN 2S albumin OS=Glycine max OX=3847 PE=1 SV=2  MTKFTILLISLLFCIAHTCSASKWQHQQDSCRKQLQGVNLTPCEKHIMEKIQGRGDDDDD  DDDDNHILRTMRGRINYIRRNEGKDEDEEEEGHMQKCCTEMSELRSPKCQCKALQKIMEN  QSEELEEKQKKKMEKELINLATMCRFGPMIQCDLSSDD | | C |
| 2 | Napin-type 2S albumin 1 | *Glycine max* (Soybean) | 155 | >tr\|Q9ZNZ4\|Q9ZNZ4_SOYBN Napin-type 2S albumin 1 OS=Glycine max OX=3847 GN=547892 PE=2 SV=1  MTKLTILLIALLFIAHTCCASKWQQHQQESCREQLKGINLNPCEHIMEKIQAGRRGEDGS  DEDHILIRTMPGRINYIRKKEGKEEEEEGHMQKCCSEMSELKSPICQCKALQKIMDNQSE  QLEGKEKKQMERELMNLAIRCRLGPMIGCDLSSDD | | C |
| 3 | Napin | *Momordica charantia* (Bitter gourd) | 140 | >tr\|Q8L694\|Q8L694_MOMCH Napin OS=Momordica charantia OX=3673 GN=napin PE=2 SV=1  MARLSSMVVLLAVALLLTDVYAYRTTITTVEVDEDNQGRHERCHHIRPREQLRSCESFLR  QSRGYLEMKGVEENQWERQQGLEECCRQLRNVEEQCRCDALQEIAREVQRQERGQEGSQM  LQKARMLPAMCGVRPQRCDF | | C |
| 4 | 2S albumin-like protein | *Picea glauca* (Pinus glauca) | 173 | >tr\|O81412\|O81412_PICGL 2S albumin-like protein OS=Picea glauca OX=3330 PE=2 SV=1  MGVFSPSTTRLTLKWFSLSVALFLLLHWGIPSVDGHEDNMYGEEIQQQRRSCDPQRDPQR  LSSCRDYLERRREEPSERCCEELQRMSPQCRCQAIQQMLDQSLSYDSFMDSDSQEDAPLN  QRRRRCREGRGREEEEAMERAAYLPNTCNVREPPRRCDIQRHSRYSMTGSSFK | | C |
| 5 | 2S albumin | *Sesamum indicum* (Oriental sesame) | 153 | >tr\|Q9AUD1\|Q9AUD1_SESIN 2S albumin OS=Sesamum indicum OX=4182 PE=2 SV=1  MAKKLALAAVLLVAMVALASATTYTTTVTTTAIDDEANQQSQQCRQQLQGRQFRSCQRYL  SQGRSPYGGEEDEVLEMSTGNQQSEQSLRDCCQQLRNVDERCRCEAIRQAVRQQQQEGGY  QEGQSQQVYQRARDLPRRCNMRPQQCQFRVIFV | | C |
| 6 | 2S albumin | *Bertholletia excelsa* (Brazil nut) | 145 | >tr\|B6EU54\|B6EU54_BEREX 2S albumin OS=Bertholletia excelsa OX=3645 PE=2 SV=1  MANISVVAAALLVLLVLGHATAFRATVTTTVVEEENQDCPEQMQRQQMLSHCRMYMRQMM  KESPYQTMPRRGMEPHMSECCEQLEGMDESCRCEGLRMMMRMMQQQEMQPRGEQMRMMMR  MAENLPSRCNLSPQRCPMAGSMAGF | | C |
| 7 | 2S albumin | Bertholletia excelsa (Brazil nut) | 146 | >tr\|B6EU55\|B6EU55_BEREX 2S albumin OS=Bertholletia excelsa OX=3645 PE=2 SV=1  MAKISVAAAALLVLMALGHATAFRATVTTTVVEEENQEECREQMQRQQMLSHCRMYMRQQ  MEESPYQTMPRRGMEPHMSECCEQLEGMDESCRCEGLRRMMRMMQQKEMQPRGEQMRRMM  RLAENIPSRCNLSPMRCPMGGSIAGF | | C |
| 8 | 2S albumin | *Ricinus communis* (Castor bean) | 258 | >sp\|P01089\|2SS_RICCO 2S albumin OS=Ricinus communis OX=3988 PE=1 SV=2  MAKLIPTIALVSVLLFIIANASFAYRTTITTIEIDESKGEREGSSSQQCRQEVQRKDLSS  CERYLRQSSSRRSPGEEVLRMPGDENQQQESQQLQQCCNQVKQVRDECQCEAIKYIAEDQ  IQQGQLHGEESERVAQRAGEIVSSCGVRCMRQTRTNPSQQGCRGQIQEQQNLRQCQEYIK  QQVSGQGPRRSDNQERSLRGCCDHLKQMQSQCRCEGLRQAIEQQQSQGQLQGQDVFEAFR  TAANLPSMCGVSPTECRF | | C |
| 9 | 2S albumin | *Moringa oleifera* (Horseradish tree) ( | 163 | >tr\|W5S2D2\|W5S2D2_MOROL 2S albumin OS=Moringa oleifera OX=3735 PE=1 SV=1  MAKLTLLLATLALLVLLANASIYRTTVELDEEPDDNQQQRCRHQFQTQQRLRACQRVIRR  WSQGGGPMEDVEDEIDETDEIEEVVEPDQARRPPTLQRCCRQLRNVSPFCRCPSLRQAVQ  SAQQQQGQVGPQQVGHMYRVASRIPAICNLQPMRCPFRQQQSS | | C |
| 10 | Sweet protein mabinlin-2 | *Capparis masaikai* (Mabinlang) | 155 | >sp\|P30233\|2SS2_CAPMA Sweet protein mabinlin-2 OS=Capparis masaikai OX=13395 PE=1 SV=3  MAKLIFLFATLALFVLLANASIQTTVIEVDEEEDNQLWRCQRQFLQHQRLRACQRFIHRR  AQFGGQPDELEDEVEDDNDDENQPRRPALRQCCNQLRQVDRPCVCPVLRQAAQQVLQRQI  IQGPQQLRRLFDAARNLPNICNIPNIGACPFRAWP | | C |
| 11 | SESA1 (Gene-AXX17_At4g31290) | *Arabidopsis thaliana* (Mouse-ear cress) | 164 | >tr\|A0A178UXL9\|A0A178UXL9_ARATH SESA1 OS=Arabidopsis thaliana OX=3702 GN=AXX17_At4g31290 PE=4 SV=1  MANKLFLVCAALALCFLLTNASIYRTVVEFEEDDATNPIGPKMRKCRKEFQKEQHLRACQ  QLMLQQARQGRSDEFDFEDDMENPQGQQQEQQLFQQCCNELRQEEPDCVCPTLKQAAKAV  RLQGQHQPMQVRKIYQTAKHLPNVCDIPQVDVCPFNIPSFPSFY | | C |
| 12 | Napin | *Brassica rapa* subsp. chinensis (Pak-choi) (Brassica chinensis) {Ngai, 2004 #31} | 184 | >tr\|D7R270\|D7R270_BRARC Napin OS=Brassica rapa subsp. chinensis OX=93385 PE=2 SV=1  MANKLFLVSATLALFFLLTNASVYRTVVEVDEDDATNPAGPFRIPKCRKEFQQAQHLKAC  QQWLHKQAMQSGSGPSWTLDGEFDFEDDMENQQQGPQQRPPLLQQCCNELHQEEPLCVCP  TLKGASKAVKQQVRQQQGQQMQGQQMQQVISRIYQTATHLPRVCNIRQVSICPFQKTMPG  PGFY | | C |
| 2S albumins with unknown antimicrobial activity | | | | | |  |
| 13 | Napin-1A | *Brassica napus* (Rape) | 110 | >sp\|P24565\|2SSI_BRANA Napin-1A OS=Brassica napus OX=3708 PE=1 SV=1  QPQKCQREFQQEQHLRACQQWIRQQLAGSPFQSGPQEGPWLREQCCNELYQEDQVCVCPT  LKQAAKSVRVQGQHGPFQSTRIYQIAKNLPNVCNMKQIGTCPFIAIPFFP | | C |
| 14 | Napin embryo-specific | *Brassica napus* (Rape) | 186 | >sp\|P09893\|2SSE_BRANA Napin embryo-specific OS=Brassica napus OX=3708 PE=2 SV=1  MANKLFLVSATLALFFLLTNASVYRTVVEVDEDDATNPAGPFRIPKCRKEFQQAQHLRAC  QQWLHKQAMQPGGGSGPSWTLDGEFDFEDDVENQQQGPQQRPPPPQQCCNELHQEEPLCV  CPTLKGASKAVRQQVRQQQGQQMQGQQMQQVISRVYQTATHLPRVCNIRQVSICPFQKTM  PGPGFY | | C |
| 15 | Napin (NAP1) | *Brassica napus* (Rape) | 180 | >sp\|P17333\|2SS4_BRANA Napin OS=Brassica napus OX=3708 GN=NAP1 PE=2 SV=1  MANKLFLVSATLAFFFLLTNASIYRTIVEVDEDDATNPAGPFRIPKCRKEFQQAQHLKAC  QQWLHKQAMQSGSGPSWTLDGEFDFEDDMENPQGPQQRPPLLQQCCNELHQEEPLCVCPT  LKGASKAVKQQVRQQQGQQGQQLQQVISRIYQTATHLPKVCNIPQVSVCPFQKTMPGPSY | | C |
| 16 | Napin-B | *Brassica napus* (Rape) | 178 | >sp\|P27740\|2SSB_BRANA Napin-B OS=Brassica napus OX=3708 GN=NAPB PE=2 SV=1  MANKLFLVSATLAFFFLLTNASIYRTVVEFDEDDATNPAGPFRIPKCRKEFQQAQHLKAC  QQWLHKQAMQSGSGPSWTLDGEFDFEDDMENPQGPQQRPPLLQQCCNELHQEEPLCVCPT  LKGASKAVKQQIQQQGQQQGKLQMVSRIYQTATHLPKVCKIPQVSVCPFQKTMPGPSY | | C |
| 17 | Allergen Sin a 1 | *Sinapis alba* (White mustard) (*B. hirta*) | 145 | >sp\|P15322\|ALL1_SINAL Allergen Sin a 1 OS=Sinapis alba OX=3728 PE=1 SV=2  PAGPFRIPKCRKEFQQAQHLRACQQWLHKQAMQSGSGPSWTLDDEFDFEDDMENPQGPQQ  RPPLLQQCCNELHQEEPLCVCPTLKGASKAVKQQVRQQLGQQGQQGPHLQHVISRIYQTA  THLPKVCNIRQVSVCPFKKTMPGPS | | C |
| 18 | Napin-1 | *Brassica napus* (Rape) | 133 | >sp\|P01091\|2SS1_BRANA Napin-1 (Fragment) OS=Brassica napus OX=3708 PE=2 SV=1  PKCRKEFQQAQHLKACQQWLHKQAMQSGGGPSWTLDGEFDFEDDMEKQGPQQRPPLHQQY  CNELQQEEPLCVCPTLRGASKAVKQQIQQQEQQQGKQQMVNRIYQTATHLPKVCNIPQVS  VCPFQKTMPGPSY | | F |
| 19 | Napin-2 (1.7S seed storage protein) | Brassica napus (Rape) | 178 | >sp\|P01090\|2SS2_BRANA Napin-2 OS=Brassica napus OX=3708 PE=2 SV=2  MANKLFLVSATLAFFFLLTNASIYRTVVEFDEDDATDSAGPFRIPKCRKEFQQAQHLRAC  QQWLHKQAMQSGGGPSWTLDGEFDFEDDMENPQGPQQRPPLLQQCCNELHQEEPLCVCPT  LKGASKAVKQQIQQQGQQQGKQQMVSRIYQTATHLPKVCNIPQVSVCPFQKTMPGPSY | | C |
| 20 | Napin-3 | *Brassica napus* (Rape) | 125 | >sp\|P80208\|2SS3_BRANA Napin-3 OS=Brassica napus OX=3708 PE=1 SV=1  SAGPFRIPKCRKEFQQAQHLRACQQWLHKQAMQSGSGPQGPQQRPPLLQQCCNELHQEEP  LCVCPTLKGASRAVKQQVRQQQGQQGQQLQQVISRIYQTATHLPKVCNIPQVSVCPFQKT  MPGPS | | C |
| 21 | Allergen Bra j 1-E | *Brassica juncea* (Asian mustard) | 129 | >sp\|P80207\|ALL1_BRAJU Allergen Bra j 1-E OS=Brassica juncea OX=3707 PE=1 SV=1  AGPFRFPRCRKEFQQAQHLRACQQWLHKQAMQSGSGPQPQGPQQRPPLLQQCCNELHQEE  PLCVCPTLKGASKAVKQQIRQQGQQQGQQGQQLQHEISRIYQTATHLPRVCNIPRVSICP  FQKTMPGPS | | C |
| 22 | *Brassica napus* napB napin | *Brassica napus* (Rape) | 178 | >tr\|Q39344\|Q39344_BRANA Brassica napus napB napin OS=Brassica napus OX=3708 PE=4 SV=1  MANKLFLVSATLAFFFLLTNASIYRTVVEFDEDDATNSAGPFRIPKCRKEFQQAQHLRAC  QQWLHKQAMQSGGGPSWTLDGEFDFEDDMENPQGPQQRPPLLQQCCNELHQEEPLCVCPT  LKGASKAVKQQIQQQGQQQGKQQMVSRIYQTRTNLPKVCNIPQVSVCPFQKTMPGPSY | | C |
| 23 | Napin | *Brassica rapa* subsp. oleifera (Turnip rape) | 184 | >tr\|Q42444\|Q42444_BRARO Napin OS=Brassica rapa subsp. oleifera OX=145471 PE=4 SV=1  MANKLFLVSATLALFFLLTNASVYRTVVEVDEDDATNPAGPFRIPKCRKEFQQAQHLKAC  QQWLHKQAMQSGSGPSWTLDGEFDFEDDVENQQQGPQQRPPLLQQCCNELHQEEPLCVCP  TLKGASKAVKQQIRQQQGQQMQGQQMQQVISRIYQTATHLPRACNIRQVSICPFQKTMPG  PGFY | | C |
| 24 | Napin | *Brassica campestris* (Field mustard) | 184 | >tr\|Q7DMU4\|Q7DMU4_BRACM Napin OS=Brassica campestris OX=3711 PE=2 SV=1  MANKLFLVSATLALFFLLTNASVYRTVVEVDEDDATNPAGPFRIPKCRKEFQQAQHLKAC  QQWLHKQAMQSGSGPSWTLDGEFDFEDDVENQQQGPQQRPPLLQQCCNELHQEEPLCVCP  TLKGASKAVKQQIRQQQGQQMQGQQMQQVISRIYQTATHLPRACNIRQVSICPFQKTMPG  PGFY | | C |
| 25 | Napin | *Brassica napus* var. napus | 180 | >tr\|Q6PZE1\|Q6PZE1_BRANA Napin OS=Brassica napus var. napus OX=138011 PE=2 SV=1  MANKLFLVSATLALFFLLTNASIYRTVVEVEEDDATNPAGPFRIPKCRKEFQQAQHLRAC  QQWLHKQAMQSGSGPSWTLDGEFDFEDDMENPQSPQQRPPLLQQCCNELHQEEPLCVCPT  LKGASKAVKQQVRQQQGQQGQQLQQVISRIYQTATHLPKVCNIPQVSVCPFQKTMPGPSY | | C |
| 26 | Napin | *Brassica napus* subsp. rapifera | 124 | >tr\|Q7M1P1\|Q7M1P1_BRANA Napin (Fragments) OS=Brassica napus subsp. rapifera OX=3709 PE=1 SV=1  PAGPFRIPKCRKEFQQAQHLRACQQWLHKQAMQSGGGPSPQGPQQRPPLLQQCCNELHQE  EPLCVCPTLKGAAKAVKQQQQQQQQQQGQQMVSRIYQTATHLPKVCNIPQVSVCPFQKTM  PGPS | | F |
| 27 | Napin 1.7S | *Brassica napus* var. napus | 106 | >tr\|Q6PZE4\|Q6PZE4_BRANA Napin 1.7S (Fragment) OS=Brassica napus var. napus OX=138011 PE=2 SV=1  GGPSWTLDGEFDFEDDMENPQGPQQRPPLLQQCCNELHQEEPLCVCPTLKGASKAVKQQI  QQQGQQQGKQQMVSRIYQTATHLPKVCNIPQVSVCPFQKTMPGPSY | | F |
| 28 | 2S seed storage protein 1 (AT2S1) | Arabidopsis thaliana (Mouse-ear cress) | 164 | >sp\|P15457\|2SS1_ARATH 2S seed storage protein 1 OS=Arabidopsis thaliana OX=3702 GN=AT2S1 PE=1 SV=1  MANKLFLVCAALALCFLLTNASIYRTVVEFEEDDATNPIGPKMRKCRKEFQKEQHLRACQ  QLMLQQARQGRSDEFDFEDDMENPQGQQQEQQLFQQCCNELRQEEPDCVCPTLKQAAKAV  RLQGQHQPMQVRKIYQTAKHLPNVCDIPQVDVCPFNIPSFPSFY | | C |
| 29 | 2S seed storage protein 2 (AT2S2) | *Arabidopsis thaliana* (Mouse-ear cress) | 170 | >sp\|P15458\|2SS2_ARATH 2S seed storage protein 2 OS=Arabidopsis thaliana OX=3702 GN=AT2S2 PE=2 SV=1  MANKLFLVCATFALCFLLTNASIYRTVVEFDEDDASNPMGPRQKCQKEFQQSQHLRACQK  LMRMQMRQGRGGGPSLDDEFDLEDDIENPQGPQQGHQILQQCCSELRQEEPVCVCPTLRQ  AARAVSLQGQHGPFQSRKIYKTAKYLPNICKIQQVGECPFQTTIPFFPPY | | C |
| 30 | 2S seed storage protein 3 (AT2S3) | *Arabidopsis thaliana* (Mouse-ear cress) | 164 | >sp\|P15459\|2SS3_ARATH 2S seed storage protein 3 OS=Arabidopsis thaliana OX=3702 GN=AT2S3 PE=1 SV=1  MANKLFLVCATLALCFLLTNASIYRTVVEFEEDDASNPVGPRQRCQKEFQQSQHLRACQR  WMSKQMRQGRGGGPSLDDEFDFEGPQQGYQLLQQCCNELRQEEPVCVCPTLKQAARAVSL  QGQHGPFQSRKIYQSAKYLPNICKIQQVGECPFQTTIPFFPPYY | |  |
| 31 | 2S seed storage protein 4 (AT2S4) | *Arabidopsis thaliana* (Mouse-ear cress) | 166 | >sp\|P15460\|2SS4_ARATH 2S seed storage protein 4 OS=Arabidopsis thaliana OX=3702 GN=AT2S4 PE=2 SV=1  MANKLFLVCAALALCFILTNASVYRTVVEFDEDDASNPIGPIQKCQKEFQQDQHLRACQR  WMRKQMWQGRGGGPSLDDEFDMEDDIENPQRRQLLQKCCSELRQEEPVCVCPTLRQAAKA  VRFQGQQHQPEQVRKIYQAAKYLPNICKIQQVGVCPFQIPSIPSYY | | C |
| 32 | 2S sulfur-rich seed storage protein 1 (BE2S1) | *Bertholletia excelsa* (Brazil nut) | 146 | >sp\|P04403\|2SS1_BEREX 2S sulfur-rich seed storage protein 1 OS=Bertholletia excelsa OX=3645 GN=BE2S1 PE=1 SV=2  MAKISVAAAALLVLMALGHATAFRATVTTTVVEEENQEECREQMQRQQMLSHCRMYMRQQ  MEESPYQTMPRRGMEPHMSECCEQLEGMDESCRCEGLRMMMMRMQQEEMQPRGEQMRRMM  RLAENIPSRCNLSPMRCPMGGSIAGF | | C |
| 33 | 2S sulfur-rich seed storage protein 2 (BE2S2) | *Bertholletia excelsa* (Brazil nut) | 154 | >sp\|P0C8Y8\|2SS2_BEREX 2S sulfur-rich seed storage protein 2 OS=Bertholletia excelsa OX=3645 GN=BE2S2 PE=3 SV=1  MAKMSVVAAALLALLVLGQATAFRTTVTTTLEEEQEENPRGRSEQQCREQMERQQQLNHC  RMYLRQQMEESPYQNPRPLRRGEEPHLDECCEQLERMDEMCRCEGLRMMLRRQREEMELQ  GEQMQRIMRKAENLLSRCNLSPQRCPMGGYTAWL | | C |
| 37 | 2S albumin seed storage protein | *Juglans nigra* (Black walnut) | 161 | >tr\|Q7Y1C2\|Q7Y1C2_JUGNI 2S albumin seed storage protein (Fragment) OS=Juglans nigra OX=16719 PE=2 SV=1  RHEARKCIFHTFSLTMARLATLAALLVALLFVANAAAFRTTITTMEIDEDIDNPRRRGEG  CQEQIQRQQNLNHCQYYLRQQSRSGGYDEDNQRQHFRQCCQQLSQIEEQCQCEGLRQAVR  RQQQQQGLRGEEMEEMVQSARDLPKECGISSQRCEIRRSWF | |  |
| 12S globulins with known antimicrobial activity | | | |  | |  |
| 38 | Glycinin G1 (GY1) | *Glycine max (Soybean)* | 495 | >sp\|P04776\|GLYG1_SOYBN Glycinin G1 OS=Glycine max OX=3847 GN=GY1 PE=1 SV=2  MAKLVFSLCFLLFSGCCFAFSSREQPQQNECQIQKLNALKPDNRIESEGGLIETWNPNNK  PFQCAGVALSRCTLNRNALRRPSYTNGPQEIYIQQGKGIFGMIYPGCPSTFEEPQQPQQR  GQSSRPQDRHQKIYNFREGDLIAVPTGVAWWMYNNEDTPVVAVSIIDTNSLENQLDQMPR  RFYLAGNQEQEFLKYQQEQGGHQSQKGKHQQEEENEGGSILSGFTLEFLEHAFSVDKQIA  KNLQGENEGEDKGAIVTVKGGLSVIKPPTDEQQQRPQEEEEEEEDEKPQCKGKDKHCQRP  RGSQSKSRRNGIDETICTMRLRHNIGQTSSPDIYNPQAGSVTTATSLDFPALSWLRLSAE  FGSLRKNAMFVPHYNLNANSIIYALNGRALIQVVNCNGERVFDGELQEGRVLIVPQNFVV  AARSQSDNFEYVSFKTNDTPMIGTLAGANSLLNALPEEVIQHTFNLKSQQARQIKNNNPF  KFLVPPQESQKRAVA | | C |
| 39 | Glycinin G2 (GY2) | *Glycine max (Soybean)* | 485 | >sp\|P04405\|GLYG2_SOYBN Glycinin G2 OS=Glycine max OX=3847 GN=GY2 PE=1 SV=2  MAKLVLSLCFLLFSGCFALREQAQQNECQIQKLNALKPDNRIESEGGFIETWNPNNKPFQ  CAGVALSRCTLNRNALRRPSYTNGPQEIYIQQGNGIFGMIFPGCPSTYQEPQESQQRGRS  QRPQDRHQKVHRFREGDLIAVPTGVAWWMYNNEDTPVVAVSIIDTNSLENQLDQMPRRFY  LAGNQEQEFLKYQQQQQGGSQSQKGKQQEEENEGSNILSGFAPEFLKEAFGVNMQIVRNL  QGENEEEDSGAIVTVKGGLRVTAPAMRKPQQEEDDDDEEEQPQCVETDKGCQRQSKRSRN  GIDETICTMRLRQNIGQNSSPDIYNPQAGSITTATSLDFPALWLLKLSAQYGSLRKNAMF  VPHYTLNANSIIYALNGRALVQVVNCNGERVFDGELQEGGVLIVPQNFAVAAKSQSDNFE  YVSFKTNDRPSIGNLAGANSLLNALPEEVIQHTFNLKSQQARQVKNNNPFSFLVPPQESQ  RRAVA | | C |
| 40 | Glycinin G3 (GY3) | *Glycine max (Soybean)* | 481 | >sp\|P11828\|GLYG3_SOYBN Glycinin G3 OS=Glycine max OX=3847 GN=GY3 PE=1 SV=1  MAKLVLSLCFLLFSGCCFAFSFREQPQQNECQIQRLNALKPDNRIESEGGFIETWNPNNK  PFQCAGVALSRCTLNRNALRRPSYTNAPQEIYIQQGSGIFGMIFPGCPSTFEEPQQKGQS  SRPQDRHQKIYHFREGDLIAVPTGFAYWMYNNEDTPVVAVSLIDTNSFQNQLDQMPRRFY  LAGNQEQEFLQYQPQKQQGGTQSQKGKRQQEEENEGGSILSGFAPEFLEHAFVVDRQIVR  KLQGENEEEEKGAIVTVKGGLSVISPPTEEQQQRPEEEEKPDCDEKDKHCQSQSRNGIDE  TICTMRLRHNIGQTSSPDIFNPQAGSITTATSLDFPALSWLKLSAQFGSLRKNAMFVPHY  NLNANSIIYALNGRALVQVVNCNGERVFDGELQEGQVLIVPQNFAVAARSQSDNFEYVSF  KTNDRPSIGNLAGANSLLNALPEEVIQQTFNLRRQQARQVKNNNPFSFLVPPKESQRRVV  A | | C |
| 41 | Glycinin G4 (GY4) | *Glycine max (Soybean)* | 563 | >sp\|P02858\|GLYG4_SOYBN Glycinin G4 OS=Glycine max OX=3847 GN=GY4 PE=1 SV=2  MGKPFTLSLSSLCLLLLSSACFAISSSKLNECQLNNLNALEPDHRVESEGGLIQTWNSQH  PELKCAGVTVSKLTLNRNGLHLPSYSPYPRMIIIAQGKGALGVAIPGCPETFEEPQEQSN  RRGSRSQKQQLQDSHQKIRHFNEGDVLVIPPGVPYWTYNTGDEPVVAISLLDTSNFNNQL  DQTPRVFYLAGNPDIEYPETMQQQQQQKSHGGRKQGQHQQEEEEEGGSVLSGFSKHFLAQ  SFNTNEDIAEKLQSPDDERKQIVTVEGGLSVISPKWQEQQDEDEDEDEDDEDEQIPSHPP  RRPSHGKREQDEDEDEDEDKPRPSRPSQGKREQDQDQDEDEDEDEDQPRKSREWRSKKTQ  PRRPRQEEPRERGCETRNGVEENICTLKLHENIARPSRADFYNPKAGRISTLNSLTLPAL  RQFQLSAQYVVLYKNGIYSPHWNLNANSVIYVTRGQGKVRVVNCQGNAVFDGELRRGQLL  VVPQNFVVAEQAGEQGFEYIVFKTHHNAVTSYLKDVFRAIPSEVLAHSYNLRQSQVSELK  YEGNWGPLVNPESQQGSPRVKVA | | C |
| 42 | Glycinin G5 (GY5) | *Glycine max (Soybean)* | 516 | >sp\|P04347\|GLYG5_SOYBN Glycinin G5 OS=Glycine max OX=3847 GN=GY5 PE=1 SV=1  MGKPFFTLSLSSLCLLLLSSACFAITSSKFNECQLNNLNALEPDHRVESEGGLIETWNSQ  HPELQCAGVTVSKRTLNRNGSHLPSYLPYPQMIIVVQGKGAIGFAFPGCPETFEKPQQQS  SRRGSRSQQQLQDSHQKIRHFNEGDVLVIPLGVPYWTYNTGDEPVVAISPLDTSNFNNQL  DQNPRVFYLAGNPDIEHPETMQQQQQQKSHGGRKQGQHRQQEEEGGSVLSGFSKHFLAQS  FNTNEDTAEKLRSPDDERKQIVTVEGGLSVISPKWQEQEDEDEDEDEEYGRTPSYPPRRP  SHGKHEDDEDEDEEEDQPRPDHPPQRPSRPEQQEPRGRGCQTRNGVEENICTMKLHENIA  RPSRADFYNPKAGRISTLNSLTLPALRQFGLSAQYVVLYRNGIYSPDWNLNANSVTMTRG  KGRVRVVNCQGNAVFDGELRRGQLLVVPQNPAVAEQGGEQGLEYVVFKTHHNAVSSYIKD  VFRVIPSEVLSNSYNLGQSQVRQLKYQGNSGPLVNP | | C |
| 12S globulins with unknown antimicrobial activity | | | | | |  |
|  | Cruciferin BnC1 (BnC1) | *Brassica napus (Rape)* | 490 | >sp\|P33523\|CRU1_BRANA Cruciferin BnC1 OS=Brassica napus OX=3708 GN=BnC1 PE=3 SV=2  MARLSSLLSFSLALLIFLHGSTAQQFPNECQLDQLNALEPSHVLKAEAGRIEVWDHHAPQ  LRCSGVSFVRYIIESKGLYLPSFFSTAKLSFVAKGEGLMGRVVPGCAETFQDSSVFQPSG  GSPSGEGQGQGQQGQGQGHQGQGQGQQGQQGQQGQQSQGQGFRDMHQKVEHIRTGDTIAT  HPGVAQWFYNDGNQPLVIVSVLDLASHQNQLDRNPRPFYLAGNNPQGQVWIEGREQQPQK  NILNGFTPEVLAKAFKIDVRTAQQLQNQQDNRGNIIRVQGPFSVIRPPLRSQRPQETEVN  GLEETICSARCTDNLDDPSNADVYKPQLGYISTLNSYDLPILRFLRLSALRGSIRQNAMV  LPQWNANANAVLYVTDGEAHVQVVNDNGDRVFDGQVSQGQLLSIPQGFSVVKRATSEQFR  WIEFKTNANAQINTLAGRTSVLRGLPLEVISNGYQISLEEARRVKFNTIETTLTHSSGPA  SYGGPRKADA | | C |
|  | Cruciferin (CRUA) | *Brassica napus (Rape)* | 488 | >sp\|P11090\|CRUA_BRANA Cruciferin OS=Brassica napus OX=3708 GN=CRUA PE=1 SV=1  MARLSSLLSFSLALLTFLHGSTAQQFPNECQLDQLNALEPSHVLKAEAGRIEVWDHHAPQ  LRCSGVSFVRYIIESKGLYLPSFFSTARLSFVAKGEGLMGRVVLCAETFQDSSVFQPSGG  SPFGEGQGQGQQGQGQGHQGQGQGQQGQQGQQGQQSQGQGFRDMHQKVEHIRTGDTIATH  PGVAQWFYNDGNQPLVIVSVLDLASHQNQLDRNPRPFYLAGNNPQGQVWIEGREQQPQKN  ILNGFTPEVLAKAFKIDVRTAQQLQNQQDNRGNIIRVQGPFSVIRPPLRSQRPQEEVNGL  EETICSARCTDNLDDPSNADVYKPQLGYISTLNSYDLPILRFLRLSALRGSIRQNAMVLP  QWNANANAVLYVTDGEAHVQVVNDNGDRVFDGQVSQGQLLSIPQGFSVVKRATSEQFRWI  EFKTNANAQINTLAGRTSVLRGLPLEVISNGYQISLEEARRVKFNTIETTLTHSSGPASY  GGPRKADA | | C |
|  | Cruciferin BnC2 (BnC2) | *Brassica napus (Rape)* | 496 | >sp\|P33524\|CRU2_BRANA Cruciferin BnC2 OS=Brassica napus OX=3708 GN=BnC2 PE=3 SV=2  MARLSSLLYFSITVLIFLHGSTAQQFPNECQLDQLNALEPSHVLKAEAGRIEVWDHHAPQ  LRCSGVSFVRYIIESQGLYLPSFLNTANVSFVAKGQGLMGRVVPGCAETFQDSSVFQPGS  GSPFGEGQGQGQQGQGQGQGQGQGKGQQGQGKGQQGQSQGQQGQGQGFRDMHQKVEHIRS  GDTIATHPGVAQWFYNNGNQPLVIVAVMDLASHQNQLDRNPSQFYLAGKNPQGQSWLHGR  GQQPQNNILNGFSPEVLAQAFKIDVRTAQQLQNQQDNRGNIVRVQGPFGVIRPPLKSQRP  QETEANGLEETICSARCTDNLDDPSNADVYKPQLGYISILNSYDLPILRVLRLSALRGSI  RQNAMVLPQWKSKSNAVLYVTDGEAQIQVVNDNGDRVFDGQVSQGQLLSIPQGFSVVKRA  TSDQFRWIEFKTNANAQINTLAGRTSVMRGLPLEVIANGYQISLEEARRVKFNTIETTLT  HSSGPASYGRPRKADA | | C |
|  | Cruciferin CRU1 (CRU1) | *Brassica napus (Rape)* | 509 | >sp\|P33525\|CRU3_BRANA Cruciferin CRU1 OS=Brassica napus OX=3708 GN=CRU1 PE=3 SV=1  MVKVPHLLVATFGVLLVLNGCLARQSLGVPPQLGNACNLDNLDVLQPTETIKSEAGRVEY  WDHNNPQIRCAGVSVSRVIIEQGGLYLPTFFSSPKISYVVQGMGISGRVVPGCAETFMDS  QPMQGQQQGQPWQGQQGQQGQQGQQGQQGQQGQQGQQGQQGQQGQQGQQQQGFRDMHQKV  EHVRHGDIIAITAGSSHWIYNTGDQPLVIICLLDIANYQNQLDRNPRTFRLAGNNPQGGS  QQQQQQQQNMLSGFDPQVLAQALKIDVRLAQELQNQQDSRGNIVRVKGPFQVVRPPLRQP  YESEQWRHPRGPPQSPQDNGLEETICSMRTHENIDDPARADVYKPNLGRVTSVNSYTLPI  LQYIRLSATRGILQGNAMVLPKYNMNANEILYCTQGQARIQVVNDNGQNVLDQQVQKGQL  VVIPQGFAYVVQSHQNNFEWISFKTNANAMVSTLAGRTSALRALPLEVITNAFQISLEEA  RRIKFNTLETTLTRARGGQPQLIEEIVEA | |  |
|  | Cruciferin CRU4 (CRU4) | *Brassica napus (Rape)* | 465 | >sp\|P33522\|CRU4_BRANA Cruciferin CRU4 OS=Brassica napus OX=3708 GN=CRU4 PE=1 SV=1  MGPTSLLSFFFTFLTLFHGFTAQQWPNECQLDQLNALEPSQIIKSEGGRIEVWDHHAPQL  RCSGFAFERFVIEPQGLYLPTFLNAGKLTFVVHGHALMGKVTPGCAETFNDSPVFGQGQG  QEQGQGQGQGQGQGFRDMHQKVEHLRSGDTIATPPGVAQWFYNNGNEPLILVAAADIANN  LNQLDRNLRPFLLAGNNPQGQQWLQGRQQQKQNNIFNGFAPQILAQAFKISVETAQKLQN  QQVNRGNIVKVQGQFGVIRPPLRQGQGGQQPQEEGNGLEETLCTMRCTENLDDPSSADVY  KPSLGYISTLNSYNLPILRFLRLSALRGSIHNNAMVLPQWNVNANAALYVTKGKAHIQNV  NDNGQRVFDQEISKGQLLVVPQGFAVVKRATSQQFQWIEFKSNDNAQINTLAGRTSVMRG  LPLEVISNGYQISPQEARSVKFSTLETTLTQSSGPMGYGMPRVEA | | C |
|  | Cruciferin PGCRURSE5 (CRURS) | *Raphanus sativus (Radish)* | 479 | >sp\|Q02498\|CRU1_RAPSA Cruciferin PGCRURSE5 OS=Raphanus sativus OX=3726 GN=CRURS PE=3 SV=1  MVKLAHLLVATFGVLLVLNGCLARQSLGVPPQLGNACNLDNLDVLQPTETIKSEAGRLEY  WDHNHPQLRCAGVSVSRLIIEQGGLYLPTFFSSPKIAYVVQGMGISGRVVPGCAETFMDS  QPMQGQGQQGQQGQQGQQQQGFRDMHQKVEHVRHGDVIAITAGSAHWIYNTGDQPLVIVC  LLDIANYQNQLDRNPRTFRLAGNNPQGGSHQQQQQQQQNMLSGFDPQVLAQALKMQLRLA  QELQNQQDNRGNIVRVKGPFQVVRPPLRQQYESEQWRHPRGPPQSPQDNGLEETICSMRT  HENIDDPARADVYKPNLGRVTSVNSYTLPILQYIRLSATRGILQGNAMVLPKYNMNANEI  LYCTQGQARIQVVNDNGQNVLDQQVQKGQLVVIPQGFAYVVQSHGNNFEWISFKTNANAM  VSTLAGRTSALRALPLEVITNAFQISLEEARRIKFNTPETTLTHARGGQPQLIEEIVEA | | C |

Sequence status F=Fragment, C=Complete.


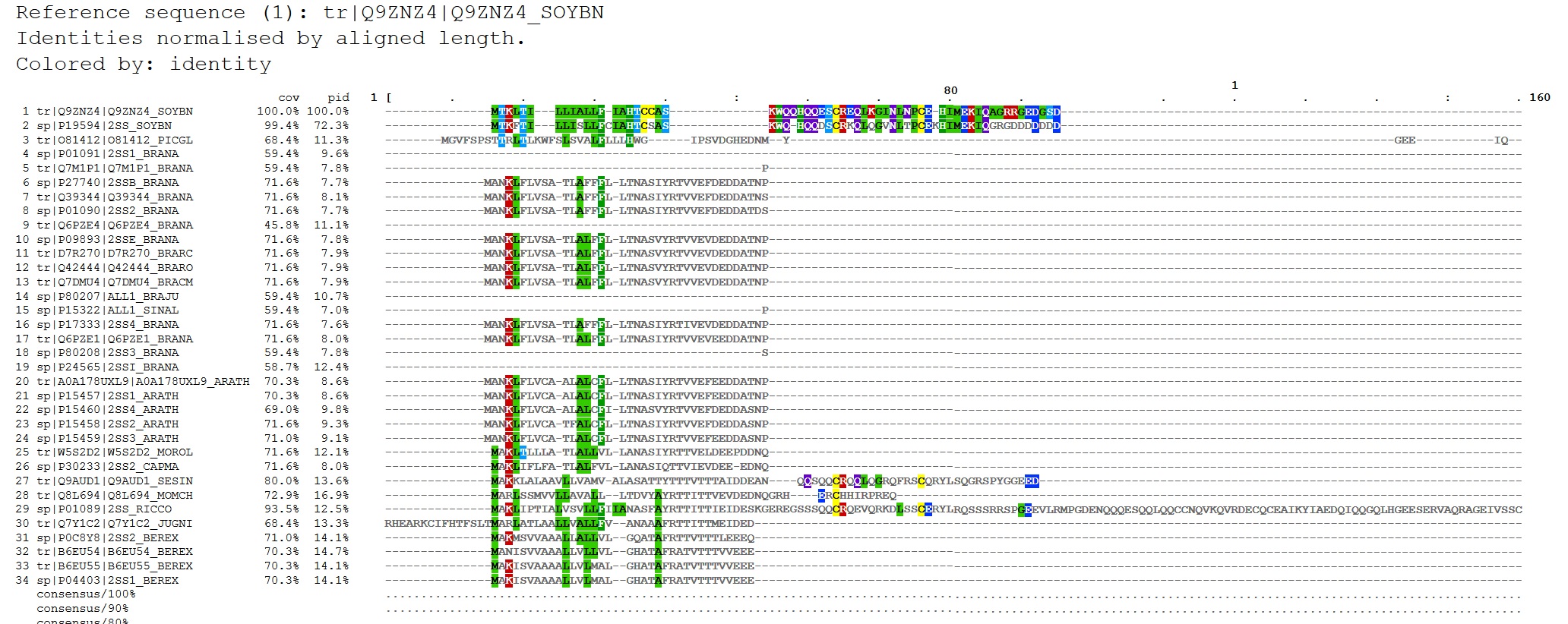


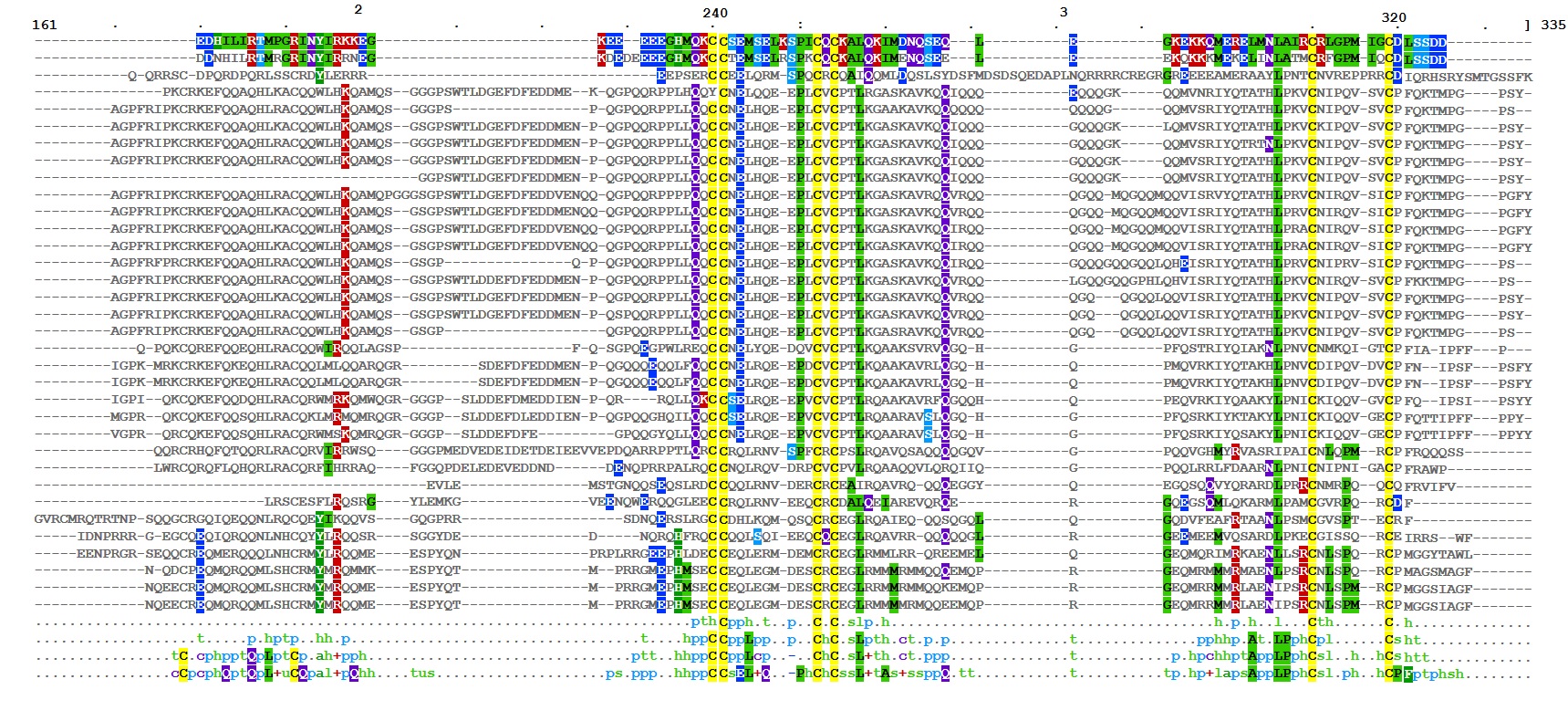


**Supplementary Figure 1a.** Multiple sequence alignment of amino acid sequences for the major rapeseed 2S albumin proteins revealed high sequence identity (pid = percent identity) and conserved sequence motifs to a series of antimicrobial proteins reported from various plant species.


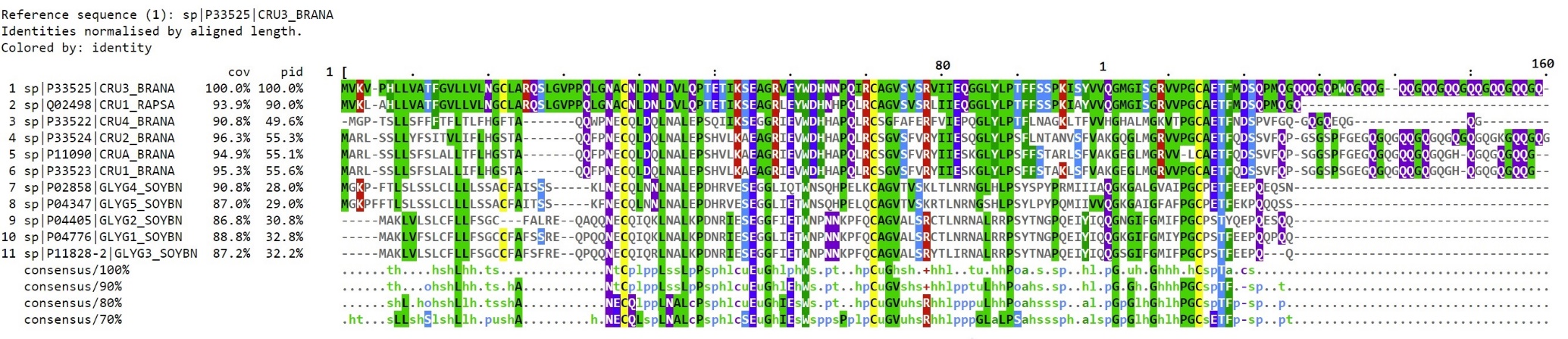


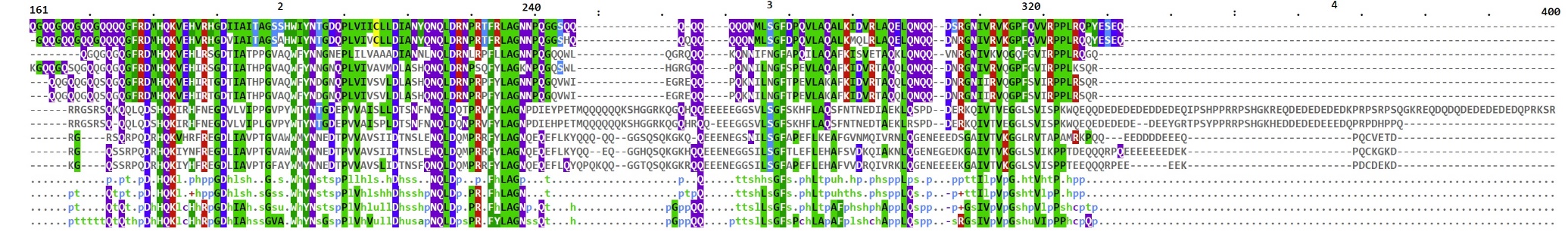


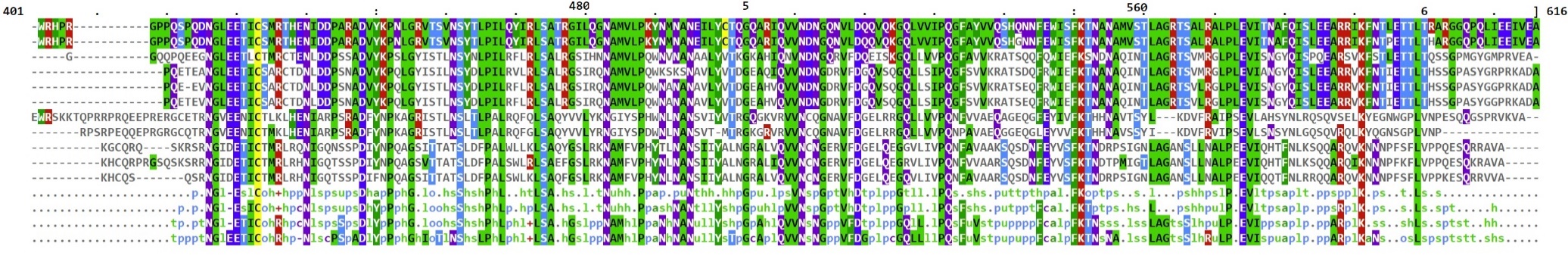


**Supplementary Figure 1b.** Multiple sequence alignment of amino acid sequences for 12S cruciferin revealed that the protein has high sequence identity (pid = percent identity) and conserved sequence motifs to a series of antimicrobial proteins reported from various plant species.
