# Supplementary material for: In Silico, Molecular Docking and In Vitro Antimicrobial Activity of the Major Rapeseed Seed Storage Proteins
Source: Front Pharmacol. 2020 Sep 8;11:1340. doi: 10.3389/fphar.2020.01340 (PMC7508056; doi:10.3389/fphar.2020.01340)

**Supplementary Figure 1A.** Multiple sequence alignment of amino acid sequences for the major rapeseed 2S albumin proteins revealed high sequence identity (pid = percent identity) and conserved sequence motifs to a series of antimicrobial proteins reported from various plant species.


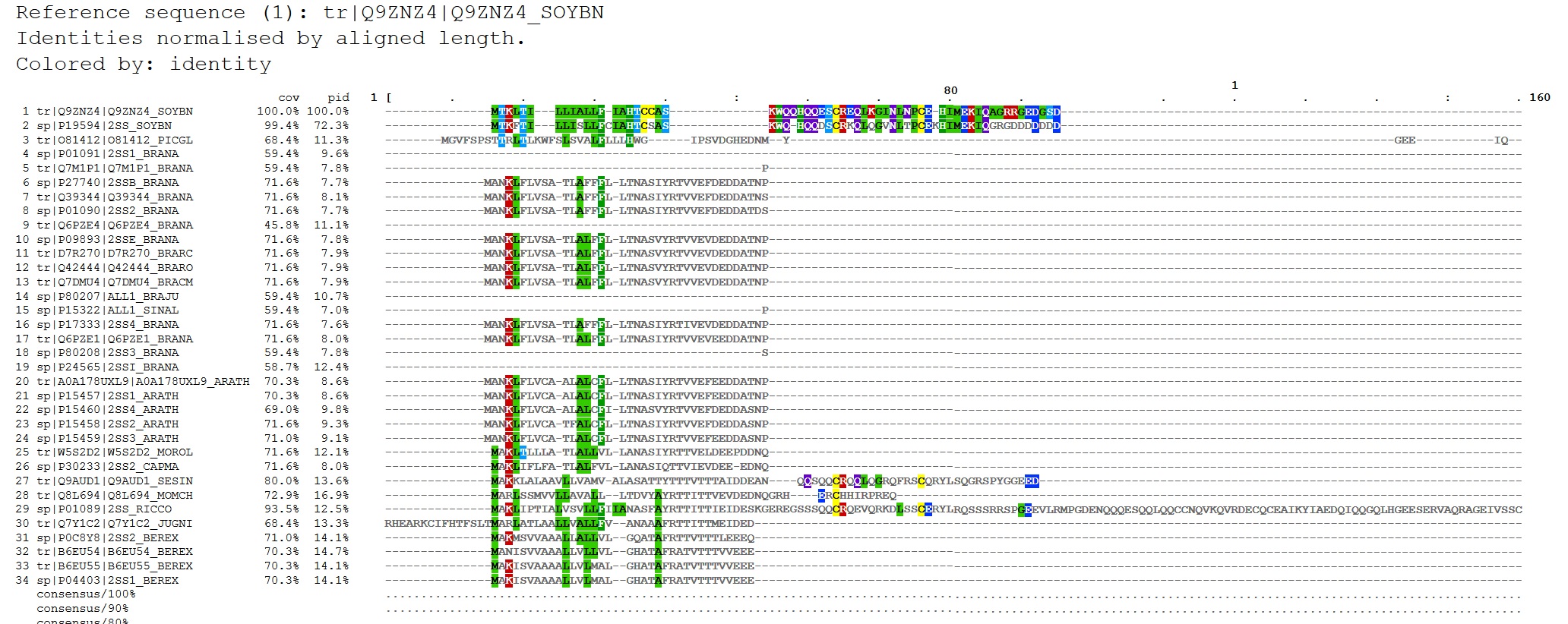


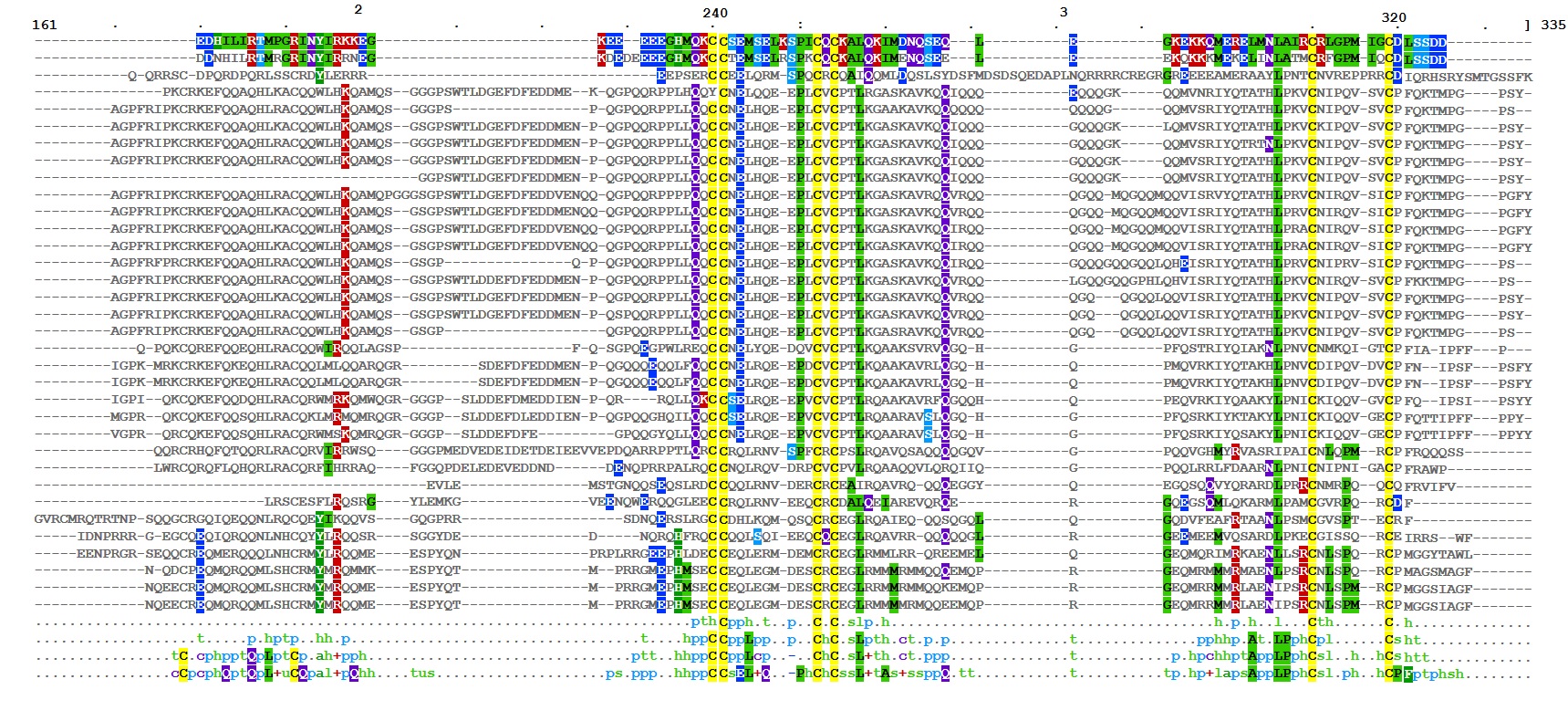


**Supplementary Figure 1B.** Multiple sequence alignment of amino acid sequences for 12S cruciferin revealed that the protein has high sequence identity (pid = percent identity) and conserved sequence motifs to a series of antimicrobial proteins reported from various plant species.

­

**
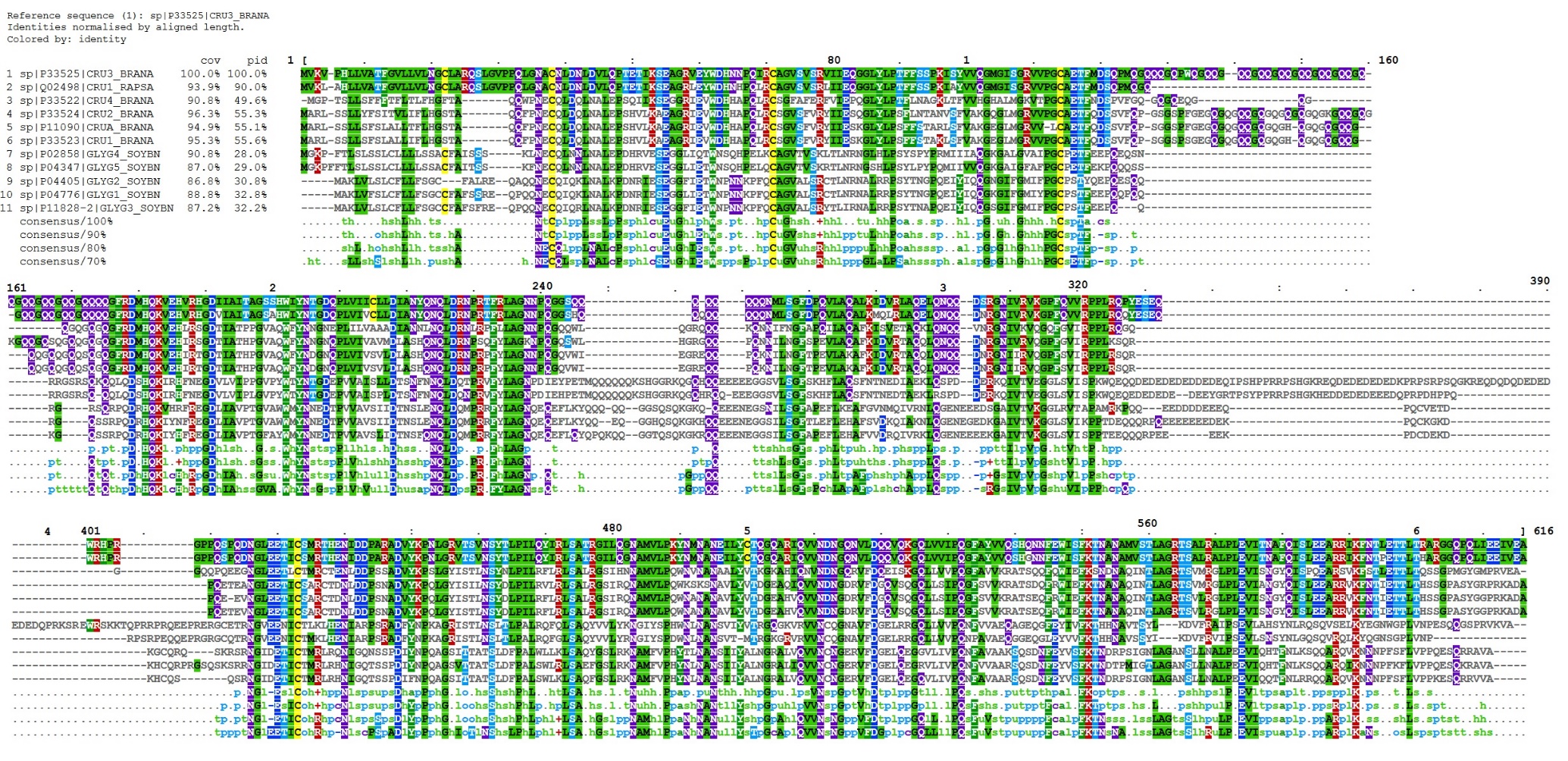
**

**Supplementary Figure 2:** A: One-Dimensional Sodium Dodecyl Sulfate-Polyacrylamide Gel Electrophoresis (1D SDS-PAGE) protein profile of purified napin and cruciferin proteins. SDS solubilised proteins were subjected to electrophoresis and the bands were stained with Coomassie Blue stain. B: Immuno-confirmation of the purity of the napin (blue arrow) and cruciferin (yellow arrow) proteins using Western blotting.


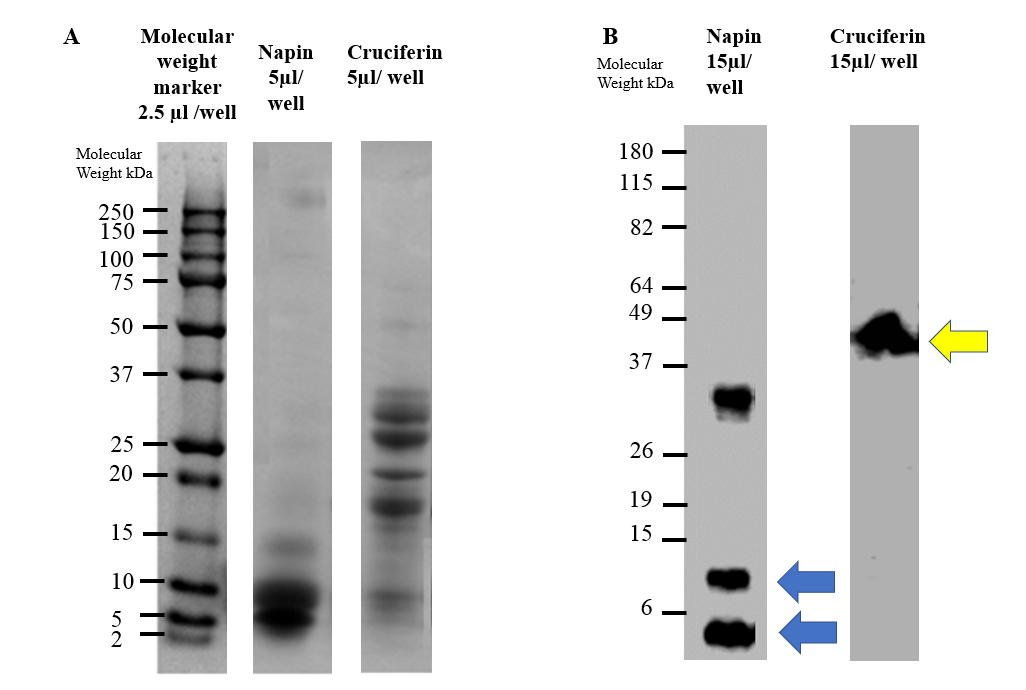

Supplement: Supplementary file 2 [file DataSheet_2.docx]
